# The epidemiological burden and societal cost of 14 respiratory conditions in the World Health Organization European region: systematic evidence map and economic analysis

**DOI:** 10.1183/23120541.01351-2025

**Published:** 2026-06-29

**Authors:** Sue Harnan, Matthew Franklin, Colin Angus, Joan B. Soriano, Ane Johannessen, Anthea Sutton, Judith Garcia-Aymerich, Amy Auer, Christopher Carroll, Emma Simpson, Pippa Powell, Lauren Anderson, Guy Joos

**Affiliations:** 1 Sheffield Centre for Health and Related Research, The University of Sheffield, Sheffield, UK; 2 Servicio de Neumología, Hospital Universitario de la Princesa, Madrid, Spain; 3 Facultad de Medicina, Universidad Autónoma de Madrid, Madrid, Spain; 4 Centro de Investigación Biomédica en Red de Enfermedades Respiratorias, Instituto de Salud Carlos III, Madrid, Spain; 5 Department of Global Public Health and Primary Care, University of Bergen, Bergen, Norway; 6 Barcelona Institute for Global Health (ISGlobal), Barcelona, Spain; 7 Medicine and Life Sciences Department, Universitat Pompeu Fabra, Barcelona, Spain; 8 European Respiratory Society, Lausanne, Switzerland; 9 European Lung Foundation, Sheffield, UK; 10 Freelance Communications Consultant, Sheffield, UK; 11 Department of Internal Medicine and Pediatrics, Faculty of Medicine and Health Sciences, Ghent University, Ghent, Belgium

## Abstract

**Introduction:**

Our systematic evidence map aimed to identify lung-related epidemiological estimates to populate the International Respiratory Coalition's Lung Facts website. We highlight important evidence gaps, suggest how they could be filled, and provide bespoke societal cost estimates to inform resource allocation.

**Methods:**

We examined 14 lung conditions across 53 World Health Organization Europe countries, seeking incidence, prevalence, mortality, years of life lost, years lived with disability, and disability-adjusted life year (DALY) estimates by age/sex. Global Burden of Disease (GBD) study estimates were obtained for nine GBD-included lung conditions: asthma, COPD, interstitial lung disease and pulmonary sarcoidosis, lower respiratory infections, lung cancer/tracheal, bronchus and lung cancer, tuberculosis, mesothelioma, COVID-19 and pulmonary arterial hypertension. Systematic searches of bibliographic databases were necessary for five non-GBD-included lung conditions: cystic fibrosis, obstructive sleep apnoea (OSA), influenza, alpha-1 antitrypsin deficiency (A1AD) and bronchiectasis. All-age country-specific DALY estimates were multiplied by gross domestic product per capita as a proxy for a country's wealth and prosperity to estimate societal costs.

**Results:**

Complete data for nine lung conditions for all countries were extracted from GBD, enabling societal cost estimation. Significant gaps were found for A1AD (only prevalence for 24 countries) and bronchiectasis (incidence, prevalence and mortality for between one and five countries), with more, but incomplete, estimates for cystic fibrosis, OSA and influenza.

**Conclusions:**

More epidemiological evidence is required for A1AD, bronchiectasis, cystic fibrosis, OSA, and influenza. Inclusion in the GDB study could help address these gaps. Lung Facts provides comprehensive lung-related epidemiological and societal cost estimates covering 53 countries to support policy-makers advocating for respiratory interventions.

## Shareable abstract

**Systematic evidence map identifies lung-related epidemiological data to populate the IRC's Lung Facts website, highlighting evidence gaps, suggesting how they could be filled and giving bespoke societal cost estimates to inform resource allocation** https://bit.ly/4klscLG

## Introduction

Respiratory diseases contribute greatly to morbidity and mortality worldwide [1]. To raise public and decision-maker awareness of lung health importance and increasing disease burden, a European Lung White Book was launched in 2003 (updated 2013) [2, 3]. The European Respiratory Society (ERS) founded the International Respiratory Coalition (IRC) in 2021, to promote lung health and improve respiratory care by setting up World Health Organization (WHO) Europe-based national respiratory strategies. An update of the White Book epidemiological and economic data was urgently needed. Thus, the “Lung Facts” website was launched in 2022 to meet this need: https://international-respiratory-coalition.org/lung-facts/

Lung Facts provides key epidemiological and economic data for 14 major chronic respiratory conditions within the 53 WHO Europe countries. The IRC updates Lung Facts every 2 years; a key data source is the Global Burden of Disease (GBD) study (www.healthdata.org/research-analysis/gbd). The GBD study collates epidemiological data from multiple sources (*e.g.* registries, epidemiological surveillance, primary scientific studies), alongside survey data on self-reported health and health-related risk behaviours. Global data are integrated into a single statistical model to estimate incidence, prevalence and mortality. A demographic model estimates years of life lost (YLL), years lived with disability (YLD), and subsequently disability-adjusted life years (DALYs). However, the GBD does not provide evidence for all lung conditions (*e.g.* cystic fibrosis, sleep apnoea) nor economic considerations (*e.g.* societal costs).

We identify and describe the epidemiology of 14 respiratory diseases in the WHO Europe region for 2010 onwards. When all-age DALY estimates are available, we subsequently estimate societal costs by monetising these DALYs using gross domestic product (GDP) per capita as a proxy for a country's wealth and prosperity. The Lung Facts website provides all identified and bespoke estimates. This article summarises our methodology and key findings, including evidence gaps with suggestions to fill these gaps.

## Methods

The 14 conditions included in Lung Facts are nine diseases reported within GBD, with a selected group of five other important respiratory diseases. These include frequent disorders (obstructive sleep apnoea (OSA), influenza), alongside less common (bronchiectasis) and rare disorders (alpha-1 antitrypsin deficiency (A1AD), cystic fibrosis). The selection of diseases was based on those that were included in the Lung White Book. This selection was reviewed and revised. They were considered to be the key respiratory conditions to include based on expert consultation.

Our methods have three elements. First, collating relevant GBD-sourced evidence. Second, collating non-GBD-sourced evidence using bibliographic searches. Third, estimating societal cost evidence not available in GBD [4]. Initial work was in 2022, with a 2024 update.

### GBD data

The nine GBD-represented conditions are asthma; COPD; interstitial lung disease and pulmonary sarcoidosis; lower respiratory infections; lung cancer/tracheal, bronchus and lung cancer; tuberculosis; mesothelioma; pulmonary arterial hypertension (2024 only); COVID-19 (2024 only). Data were downloaded from the GBD website [5] for 2015 and 2019 (in 2022), then 2021 (in 2024) (table 1). The GBD study focuses on epidemiological estimates: incidence, prevalence, mortality, YLL, YLD, and DALYs. Absolute population counts and estimated rates per 100 000 people were extracted. Estimates were stratified by age, sex, country, region and across Europe. Data samples were checked manually against the GBD website to ensure fidelity.

**TABLE 1:** Inclusion criteria for Global Burden of Disease (GBD) data and bibliographic searches for non-GBD data to identify systematic reviews and primary studies

	Inclusion criteria
**Population**	
GBD data	<5 years; 5–9 years; 5-year increments to 90–94 years; ≥95 years, age-standardised
Bibliographic searches	Data by any age range, *e.g.* everyone, adults, children, *etc.*
**Exposure and study design**	
1) GBD data	Asthma; COPD; interstitial lung disease and pulmonary sarcoidosis; lower respiratory infections; lung cancer/tracheal, bronchus and lung cancer; tuberculosis; mesothelioma; pulmonary arterial hypertension^#^; COVID-19^#^
2) Bibliographic searches	a) Systematic reviews^¶^: cystic fibrosis; OSA; influenza b) Primary studies: alpha-1 antitrypsin deficiency; bronchiectasis; pulmonary hypertension^#^
**Outcomes**	Rates and absolute numbers of: incidence; prevalence; DALYs; YLL; mortality Any type of reporting of the outcome. For example, rates may be age-standardised, reported per capita, reported as a ratio (1 case per X head of population) reported by sex, *etc*.
**Settings**	
WHO European Region	
Central Asia	Armenia, Azerbaijan, Georgia, Kazakhstan, Kyrgyzstan, Tajikistan, Türkiye, Turkmenistan, Uzbekistan
Eastern Europe	Albania, Bosnia and Herzegovina, Bulgaria, Croatia, Czech Republic, Hungary, Macedonia, Montenegro, Poland, Romania, Serbia, Slovakia, Slovenia
Central Europe	Belarus, Estonia, Latvia, Lithuania, Moldova, Russia, Ukraine
Western Europe	Andorra, Austria, Belgium, Cyprus, Denmark^+^, Finland, France, Germany, Greece, Greenland^+^, Iceland, Ireland, Israel, Italy, Luxembourg, Malta, Monaco, Netherlands, Norway, Portugal, San Marino, Spain, Sweden, Switzerland, United Kingdom

WHO: World Health Organization; OSA: obstructive sleep apnoea; DALYs: disability-adjusted life years; YLL: years of life lost. ^#^: pulmonary arterial hypertension and COVID-19 were added to the GBD study in 2024. Therefore, pulmonary hypertension was dropped from search 2b in 2024, and both conditions were added to search 1; ^¶^: where no systematic reviews were available, the criteria were widened to include nonsystematic reviews, if these provided data of use to the review; ^+^: Greenland is part of the Kingdom of Denmark, but is reported separately by the GBD study.

### Non-GBD data: bibliographic searches

Focused bibliographic searches for the five conditions not within GBD (cystic fibrosis, OSA, influenza, A1AD, bronchiectasis) sought systematic reviews for commonly studied conditions, and any-design studies for less commonly studied conditions (table 1, Exposure and study design). Searches were run in 2022 in MEDLINE and updated in 2024 with MEDLINE, Embase and the Cochrane Library. Searches were structured around terms for the conditions and terms for the outcomes. The bibliographic searches were necessarily focussed due to time and resource constraints. To mitigate the risk of missing important studies (see also Discussion, Collated data: strengths and limitations), we selected MEDLINE as the principal database, as it has been shown to retrieve >90% [6] of studies that are findable using bibliographic database searches. We used additional search techniques (*i.e.* citation chasing, reference list checking, focused searches on Google scholar, and consultation with experts) to identify additional studies (supplementary file S1). Search results were exported into EndNote and duplicates removed. Pulmonary hypertension was included in the 2022 searches, but not in 2024 since it was added to the GBD study.

Table 1 details the eligibility criteria and population, exposure, study design, outcomes, and setting. Studies were selected for inclusion by two reviewers. Disagreements were resolved through discussion between the reviewers, or with topic experts. Data could be expressed in any format, *e.g.* absolute numbers, ratios, per capita, per 100 000 people. Where possible, rate data were converted to rate per 100 000 people.

In 2022, data were extracted by one reviewer and checked by a second (supplementary file S1). In 2024, summary data were extracted into a table by one reviewer to create an evidence map. ERS disease experts used the evidence-map to decide if a Lung Facts update was required. No 2024 update data were deemed superior to the existing 2022 data; thus, full data extraction with double checking was not performed. Links to new studies are provided within Lung Facts.

### Societal cost estimation

When operationalising health-related metrics (*e.g.* DALYs or quality-adjusted life years (QALYs)) to inform resource allocation, applying a monetary value to (*i.e.* monetising) those health-related metrics is needed to determine the “opportunity cost” from any financial investment, *i.e.* what is foregone when investing in health-related outputs because the resources are no longer available to spend elsewhere. We refer to monetised health-related metrics as a societal cost, *i.e.* the health burden's monetary value. It is important to recognise that these monetised health-related metrics represent the monetised burden-of-illness (*i.e.* monetised DALYs), not the cost of illness, such as the amount of money the healthcare system pays to prevent/treat such conditions.

DALY monetisation using GDP stems from the WHO's Choosing Interventions that are Cost Effective (WHO-CHOICE) initiative, whereby 1–3×GDP per capita was suggested as a country-specific cost-effectiveness threshold (*e.g.* the amount of money a country is willing to pay to prevent one DALY) for cost-effectiveness analysis [7–10]. Robinson *et al*. [10] describes the GDP-based threshold rationale. In essence, GDP is a standard measure of value created through goods and services in a country during a certain period: it measures the income earned from production, or the total amount spent on final goods and services (less imports) [11]. GDP guides policy-makers, investors and businesses in strategic decision-making given an economy's activity, with GDP per capita being the GDP per person in a country's population. Thus, the WHO report suggested that a DALY prevented should be equivalent to the per-capita income, or extra market income, created from an intervention's outcome. However, benefits may be (up to three times) higher due to non-income-based benefits (*e.g.* reducing pain/suffering) [7]. Therefore, without a defined and/or evidence-based DALY monetary value, 1–3×GDP per capita could be used. More recently, WHO-CHOICE moved away from using any defined cost-effectiveness threshold, not just GDP-based thresholds [12].

Franklin *et al.* [13] rationalised why, in the absence of a defined or evidence-based DALY monetary value, using 1×GDP per capita as a benchmarked value (*i.e.* a reference point) of what countries should aim to invest in health-related outcomes could still have many benefits for transparent and consistent decision-making, citing others who debate for and against this point. Franklin *et al.* [13] subsequently suggested using 1×GDP per capita specifically, as it better aligns with monetised QALY valuations as a similar, widely used health-related metric.

Thus, we use GDP per capita to estimate the resources available in each country that could be invested in lung interventions (*e.g.* preventative strategies and medical care) to address the DALY burden of lung condition(s). That is, 1×GDP per capita is multiplied by the all-age DALY burden at a population-level or rate per 100 000 people to estimate the societal cost, to be used as a reference point for negotiation with policy-makers investing in lung interventions. GDP per capita is obtained from the WHO Global Health Expenditure Database [14], presented in 2021 values based on United States Dollars (USD$$2021), international dollars (Int$$2021), Euros (EUR€2021), and National Currency Units (NCU2021).

## Results

**FIGURE 1:**
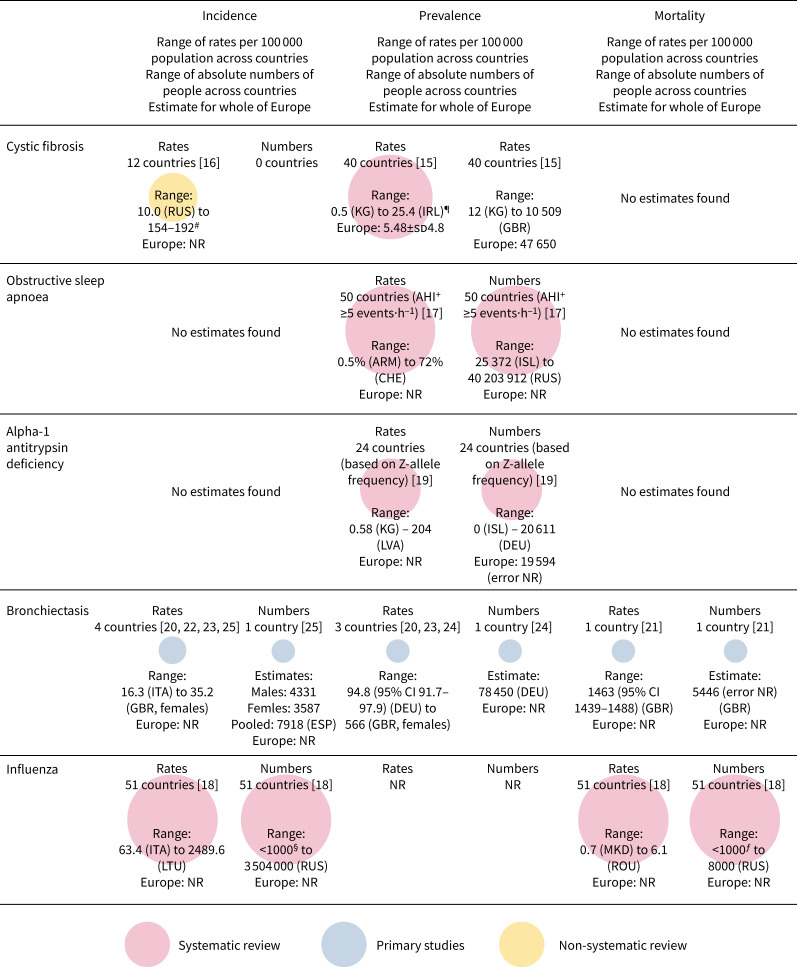
Evidence map for conditions not covered by the Global Burden of Disease Study. RUS: Russia; NR: not reported; KG: Kyrgyzstan; IRL: Ireland; GBR: United Kingdom of Great Britain and Northern Ireland; AHI: apnoea–hypopnoea index; ARM: Armenia; CHE: Switzerland; ISL: Iceland; LVA: Latvia; DEU: Germany; ITA: Italy; ESP: Spain; LTU: Lithuania; MKD: Macedonia; ROU: Romania. ^#^: data were reported as ratios. The upper ratio was reported as being between 1:5200 and 1:6500 and the lower as <1:7000. Reported for Czech Republic, Poland, Slovakia, Denmark, Netherlands; ^¶^: data were reported as rate per 10 000, and converted to rate per 100 000; ^+^: data were also reported for AHI ≥15 events·h^−1^; ^§^: reported for Andorra, Greenland and Iceland; ^ƒ^: reported for Armenia, Azerbaijan, Georgia, Kazakhstan, Kyrgyzstan, Tajikistan, Turkmenistan, Uzbekistan, Albania, Bosnia and Herzegovina, Bulgaria, Croatia, Czech Republic, Hungary, Macedonia, Montenegro, Poland, Romania, Serbia, Slovakia, Slovenia, Belarus, Estonia, Latvia, Lithuania, Moldova, Andorra, Austria, Belgium, Cyprus, Denmark, Finland, France, Germany, Greece, Greenland, Iceland, Ireland, Israel, Italy, Luxembourg, Malta, Netherlands, Norway, Portugal, Spain, Sweden, Switzerland.

To complement Lung Facts, we describe example GBD and non-GBD epidemiological estimates, and bespoke societal cost estimates, in tables 2 and 3 and figure 1. Lung Facts provides all estimates by country, lung condition, age and sex: https://international-respiratory-coalition.org/lung-facts

**TABLE 2:** Asthma Global Burden of Disease (GBD) 2021: age-standardised estimates per 100 000 people for 54 countries and World Health Organization (WHO) European subregions

	Incidence (95% UI)	Prevalence (95% UI)	DALYs (95% UI)	YLL (95% UI)	Mortality (95% UI)
**WHO European subregions**	535.4 (434.1–673.4)	4607.1 (3907.7–5378.7)	204.1 (137.5–292.3)	22.6 (21.1–24.2)	0.9 (0.9–1)
Central Asia	409.3 (338.4–509.6)	2577.2 (2227.1–2979.2)	198.8 (158.9–245.6)	97.9 (84.5–114)	4.3 (3.8–5.1)
Armenia	322.9 (250.7–419)	1753.8 (1411.3–2210)	77.6 (49.4–111.4)	7.6 (6.7–8.7)	0.4 (0.3–0.4)
Azerbaijan	379.9 (310.9–480)	2247.7 (1909.2–2633.4)	168.2 (128.9–222.2)	79.9 (58.8–116.1)	3.6 (2.7–4.8)
Georgia	370.4 (296.8–470.7)	2016.7 (1680.7–2434.5)	114.8 (84.7–157.4)	34.5 (29.6–40.8)	1.6 (1.4–1.9)
Kazakhstan	284.4 (233.7–357.9)	1599.8 (1361–1888.6)	250.4 (200.8–310.8)	187.9 (146.1–241.2)	8.6 (6.7–11.3)
Kyrgyzstan	421.5 (343.4–536.1)	2572.9 (2149.4–3076)	134.6 (96.5–185.3)	32.8 (26.4–40)	1.3 (1.1–1.6)
Tajikistan	378.7 (311.7–476.5)	2170.6 (1869.8–2590.1)	212.5 (156.8–273.4)	127.5 (90.6–175.6)	6.6 (4.7–8.8)
Türkiye	711.3 (593.5–869.4)	4888.7 (4199.5–5680.4)	249.1 (176.9–342)	58.4 (46.8–71.9)	3.1 (2.4–4)
Turkmenistan	318.9 (254.1–407.4)	1750 (1442.3–2138.7)	118.1 (86.1–159.1)	48.4 (32.7–70.5)	1.6 (1.1–2.2)
Uzbekistan	532.7 (441.5–652.7)	3791.6 (3314.2–4312.9)	237.1 (181.8–307.1)	89.5 (75.6–107.7)	3.9 (3.4–4.7)
Central Europe	898.7 (725.3–1136.9)	5643 (4722.7–6691.6)	239.1 (156.5–350.5)	15.7 (14.4–17.3)	0.8 (0.7–0.9)
Albania	471.2 (382.9–589.6)	2687.2 (2244–3207.9)	164.3 (117.4–221.4)	58 (40.8–82.4)	3.4 (2.3–4.8)
Bosnia and Herzegovina	746.3 (605.8–943.4)	4972.2 (4133.3–6036.3)	214.3 (139.4–308)	18.6 (14.3–24)	0.9 (0.7–1.2)
Bulgaria	587.2 (470.3–744.4)	3606.6 (2945.8–4410.9)	150.2 (94.5–221.2)	7.3 (6.2–8.5)	0.3 (0.3–0.4)
Croatia	639.3 (520.1–806.4)	4070.4 (3362.3–4977)	172.9 (111.9–249.8)	12.3 (10–15)	0.7 (0.6–0.9)
Czech Republic	517.4 (413.3–666)	3043.3 (2488.7–3715.8)	130.5 (82.5–189.1)	9.6 (8.4–10.8)	0.5 (0.4–0.6)
Hungary	544.2 (437.9–696.5)	3282.8 (2700.8–3982.4)	140.1 (89.3–205.9)	9.9 (8.6–11.1)	0.5 (0.4–0.5)
Macedonia	796.4 (663.8–986.2)	5654.9 (4836.5–6652.9)	261.4 (180.7–362.7)	41.6 (30–63.9)	2.3 (1.8–3.4)
Montenegro	591.6 (473.4–753.5)	3594.6 (2887.7–4497.3)	149.1 (93.8–218.8)	6.1 (4.7–7.7)	0.3 (0.3–0.4)
Poland	1467.9 (1163.4–1879.8)	9329.1 (7839.4–11 200.3)	384.9 (251.4–567.2)	15.9 (14.5–17)	0.8 (0.7–0.8)
Romania	699.7 (560.4–881.4)	4526.6 (3745.4–5513.6)	192.2 (123.1–277.7)	12.7 (11.1–14.2)	0.6 (0.5–0.7)
Serbia	536.9 (434.5–675.9)	3234.9 (2734.7–3871.5)	163.7 (113.7–227.2)	35.9 (29.1–44.2)	1.9 (1.5–2.3)
Slovakia	515.6 (414.6–652)	3060.8 (2533.7–3742.6)	131.1 (83.7–192.8)	9.6 (7.8–12.5)	0.5 (0.4–0.6)
Slovenia	777.4 (631.1–977.5)	5374.8 (4469.3–6479.2)	218 (138.1–318.9)	5.7 (4.9–6.5)	0.3 (0.3–0.4)
Eastern Europe	460.1 (360.8–601.7)	2622 (2125–3234)	116.5 (76.3–169.5)	12.5 (11.6–13.4)	0.5 (0.4–0.5)
Belarus	627.8 (494.7–793)	4210.1 (3487.4–5059.4)	172.8 (111.2–252)	7.2 (5.9–8.6)	0.2 (0.2–0.3)
Estonia	463 (372.9–591.7)	2789.3 (2330.5–3387.6)	121.8 (80.1–174.5)	11.6 (10.1–13.1)	0.6 (0.5–0.6)
Latvia	510.1 (408.5–647.2)	3094.6 (2578.2–3753.2)	138.1 (91.7–198.7)	15.5 (13.4–17.8)	0.7 (0.6–0.8)
Lithuania	476.5 (381.4–609.4)	2819.6 (2331.8–3408.3)	121.6 (77.8–176)	10 (8.7–11.2)	0.4 (0.4–0.5)
Moldova	464.5 (366.5–598.2)	2692.8 (2203.9–3279.2)	117.9 (76–172.1)	10.7 (9.4–12.2)	0.4 (0.3–0.4)
Russia	431.1 (337.9–565.9)	2418.9 (1960.8–2967.8)	111 (74.2–160.6)	15.1 (14–16.2)	0.6 (0.5–0.6)
Ukraine	534.2 (408.2–709.5)	2970.9 (2345.2–3690.8)	123.9 (76.2–182.6)	5.4 (3.9–7.1)	0.2 (0.1–0.2)
Western Europe	498.8 (404.7–632.6)	5886.8 (4944–6943.6)	248.3 (161.3–359.5)	16.4 (15.5–17)	0.7 (0.6–0.7)
Andorra	494 (401.2–626.5)	6536.6 (5337.2–7774.3)	274.9 (177.1–406.9)	17.3 (12.1–23)	0.6 (0.4–0.8)
Austria	416.6 (339.9–532.7)	4788.8 (3942.2–5796.4)	203.1 (131.4–297.4)	14.1 (13.2–15)	0.5 (0.5–0.6)
Belgium	407 (329.6–530.6)	4618.8 (3855.3–5517.6)	197.9 (128.9–283.9)	16 (14.9–17.1)	0.6 (0.5–0.7)
Cyprus	524.6 (417.5–661.2)	7411.9 (6003.5–9009.1)	327.1 (214.7–474.7)	36.5 (30.5–43.9)	2 (1.7–2.5)
Denmark^#^	460.5 (371.7–582.5)	4631.3 (3772.2–5507.2)	201.3 (132.7–287.3)	18.6 (17.1–20)	0.8 (0.7–0.8)
Finland	462.6 (378.3–586.9)	6163 (5111.4–7331)	255.5 (165.8–365.9)	14 (12.7–15)	0.7 (0.6–0.7)
France	484.6 (392.3–611.2)	6244.6 (5192.7–7435.8)	261.2 (167.8–381.1)	15.6 (14.5–16.6)	0.6 (0.6–0.7)
Germany	388.8 (315.8–498.4)	4262.2 (3529.5–5050.3)	183.5 (119–260.6)	15.5 (14.5–16.5)	0.6 (0.5–0.6)
Greece	411.5 (328.4–522.7)	4781.9 (3914.3–5721.5)	194.3 (122.7–283.2)	6 (5.7–6.4)	0.2 (0.2–0.2)
Greenland^#^	1067.7 (865.6–1350.2)	7632.2 (6612.9–8851)	350.6 (241.7–494.6)	51.6 (42.5–62.4)	1.5 (1.3–1.8)
Iceland	634.6 (511.8–817.3)	7330.5 (5957.6–8914.1)	306.5 (193.1–453.2)	16.6 (15–18.2)	0.6 (0.6–0.7)
Ireland	525.5 (417.6–670.5)	6914.5 (5760.1–8351.5)	288.1 (181–418.3)	15.4 (14.1–16.5)	0.6 (0.5–0.7)
Israel	365.5 (294.4–472.9)	3765.6 (3130.6–4472.4)	169.5 (112.3–240.2)	20.6 (18.6–22)	1 (0.8–1.1)
Italy	415.1 (323.4–540.1)	3722.9 (2995.2–4569)	152.4 (96.5–227.4)	5.6 (5.1–5.9)	0.3 (0.2–0.3)
Luxembourg	514.3 (417.4–662)	7102.3 (5964–8441.9)	299.8 (194.3–439.5)	20 (17.9–22.2)	0.8 (0.8–0.9)
Malta	502.1 (404–644.3)	6700.3 (5456–8082)	277 (175.6–410.3)	13.5 (11.9–15.3)	0.6 (0.5–0.6)
Monaco	474.6 (376.4–605.8)	6054.7 (4991–7148.9)	243.7 (151.6–370.9)	4.7 (3.6–6)	0.2 (0.1–0.2)
Netherlands	339 (280.7–419.6)	6507.1 (5455.3–7766.1)	265.9 (169.1–388.9)	9.7 (9–10.4)	0.4 (0.4–0.5)
Norway	644.3 (511–823.6)	6050.4 (4949.5–7293.2)	255.7 (167.2–375.3)	16.7 (15.6–17.5)	0.8 (0.7–0.9)
Portugal	621.8 (514.9–792.4)	9465.4 (7816.9–11 402.8)	386.8 (246.2–572.9)	15.8 (14.7–16.7)	0.6 (0.6–0.7)
San Marino	474.2 (382.8–600)	5999.9 (4970.9–7191.1)	243.4 (154.8–364.1)	7 (4.5–9.7)	0.3 (0.2–0.4)
Spain	402.7 (322.2–512.9)	4332.6 (3602.4–5189.6)	187 (122.3–264.8)	16.3 (14.4–17.5)	0.9 (0.7–1)
Sweden	679.6 (541.4–872.6)	7522.6 (6140.8–9106.1)	309.1 (199–459.2)	12.5 (11.2–13.9)	0.6 (0.5–0.7)
Switzerland	465.8 (375.8–604.2)	5655.5 (4527–6839.2)	235.2 (149.9–345)	12.4 (11.4–13.4)	0.5 (0.5–0.6)
United Kingdom	793.7 (643.1–999)	10 029.8 (8450.2–11 747.4)	427.7 (282.7–620.1)	33.1 (31.5–34.1)	1.3 (1.1–1.3)

95% UI: 95% uncertainty interval; DALYs: disability-adjusted life years; YLL: years of life lost. **^#^**: Greenland is part of the Kingdom of Denmark, but is reported separately by the GBD study.

**TABLE 3:** Asthma societal cost examples based on Global Burden of Disease (GBD) 2021 disability-adjusted life year (DALY) rates and gross domestic product (GDP) per capita by 53 countries and World Health Organization (WHO) European region

	NCU	DALY rates per 100 000 people,	GDP per capita (2021)				Societal cost per 100 000 people (2021)^#^			
		Mean (95% CI)	Int$^¶^	USD	EUR	NCU	Int$^¶^	USD	EUR	NCU
**Albania**	ALL	191.2 (142.7–250)	15 572	6395	5407	662 007	2 974 258	1 221 439	1 032 720	126 443 404
**Andorra**	EUR	280.6 (188.7–401.3)	58 885	42 066	35 567	35 567	16 487 824	11 778 492	9 958 644	9 958 644
**Armenia**	AMD	72.5 (48.6–102.1)	15 592	4967	4199	2 501 981	1 122 659	357 589	302 340	180 142 615
**Austria**	EUR	206 (138.9–291.7)	60 118	53 777	45 468	45 468	12 384 331	11 077 984	9 366 369	9 366 369
**Azerbaijan**	AZN	161.9 (123.4–214.1)	15 585	5296	4478	9004	2 509 216	852 730	720 978	1 449 641
**Belarus**	BYN	177.6 (118.3–251.5)	21 160	7121	6021	18 078	3 745 273	1 260 403	1 065 663	3 199 785
**Belgium**	EUR	199.8 (136.6–277.3)	59 035	51 438	43 490	43 490	11 747 995	10 236 127	8 654 584	8 654 584
**Bosnia and Herzegovina**	BAM	223.5 (155.9–306.8)	17 701	7230	6113	11 956	3 947 385	1 612 331	1 363 216	2 666 161
**Bulgaria**	BGN	138.9 (92.8–194.2)	27 948	12 156	10 278	20 099	3 856 862	1 677 509	1 418 324	2 773 594
**Croatia**	EUR	183 (125.2–253.3)	33 381	17 094	14 453	14 453	6 108 703	3 128 256	2 644 922	2 644 922
**Cyprus**	EUR	354.3 (236.8–499.1)	44 610	31 709	26 810	26 810	15 791 768	11 224 953	9 490 630	9 490 630
**Czech Republic**	CZK	122.9 (83.4–169.1)	44 783	26 331	22 263	570 813	5 463 524	3 212 413	2 716 076	69 639 227
**Denmark^+^**	DKK	202.6 (140.3–279.1)	65 069	68 202	57 664	428 794	13 144 001	13 776 822	11 648 220	86 616 440
**Estonia**	EUR	129.1 (91.1–177)	43 505	27 962	23 642	23 642	5 612 158	3 607 080	3 049 764	3 049 764
**Finland**	EUR	280.3 (191.6–389)	54 756	53 538	45 266	45 266	15 331 708	14 990 728	12 674 570	12 674 570
**France**	EUR	271.4 (183.9–380.4)	51 434	43 719	36 964	36 964	13 938 692	11 847 840	10 017 278	10 017 278
**Georgia**	GEL	127.2 (101.1–163.6)	16 790	4956	4190	15 967	2 132 325	629 435	532 183	2 027 786
**Germany**	EUR	186.8 (129.1–256.3)	58 813	51 229	43 314	43 314	10 939 237	9 528 564	8 056 344	8 056 344
**Greece**	EUR	193.1 (125.4–275.5)	31 185	20 122	17 013	17 013	6 018 737	3 883 519	3 283 492	3 283 492
**Hungary**	HUF	134 (90.9–186.5)	36 694	18 732	15 838	5 678 391	4 917 050	2 510 069	2 122 248	760 904 392
**Iceland**	ISK	302.7 (196.6–436.3)	58 524	68 999	58 338	8 762 068	17 674 220	20 837 612	17 618 075	2 646 144 575
**Ireland**	EUR	285.6 (185.1–404.9)	107 142	100 709	85 149	85 149	30 535 405	28 702 112	24 267 462	24 267 462
**Israel**	ILS	173.2 (116.3–241.9)	46 365	54 890	46 409	177 306	8 021 149	9 496 016	8 028 824	30 674 011
**Italy**	EUR	154.3 (102.7–222.9)	46 593	35 694	30 179	30 179	7 175 390	5 496 821	4 647 529	4 647 529
**Kazakhstan**	KZT	240.2 (192.3–296)	28 393	10 268	8682	4 373 284	6 814 272	2 464 357	2 083 599	1 049 588 067
**Kyrgyzstan**	KGS	128.5 (90.1–179.8)	5548	1339	1132	113 334	710 122	171 393	144 911	14 506 805
**Latvia**	EUR	142.9 (102–194.8)	34 887	21 001	17 756	17 756	4 953 944	2 982 144	2 521 384	2 521 384
**Lithuania**	EUR	118.7 (82.3–164)	43 866	23 759	20 088	20 088	5 176 246	2 803 555	2 370 389	2 370 389
**Luxembourg**	EUR	311.6 (208.1–448.1)	132 583	134 713	113 899	113 899	41 233 169	41 895 685	35 422 549	35 422 549
**Macedonia**	MKD	286.2 (202.5–390.1)	18 006	6573	5558	342 511	5 149 786	1 879 961	1 589 496	97 958 192
**Malta**	EUR	293.1 (195.3–419.4)	50 230	34 406	29 090	29 090	14 717 393	10 080 814	8 523 268	8 523 268
**Moldova**	MDL	108.8 (73.1–152.3)	15 572	5293	4475	93 575	1 681 737	571 597	483 282	10 106 120
**Monaco**	EUR	240.1 (154.9–348.9)	238 509	234 317	198 114	198 114	57 242 067	56 236 100	47 547 284	47 547 284
**Montenegro**	EUR	136.7 (88.2–197.1)	23 111	9335	7893	7893	3 143 127	1 269 576	1 073 418	1 073 418
**Netherlands**	EUR	276.6 (180.5–396.8)	63 562	57 898	48 953	48 953	17 543 085	15 979 966	13 510 965	13 510 965
**Norway**	NOK	259.3 (176.2–365.5)	82 124	90 940	76 890	781 178	21 270 165	23 553 569	19 914 400	202 325 157
**Poland**	PLN	368.2 (249.6–525)	38 148	18 006	15 224	69 538	14 038 408	6 626 198	5 602 411	25 589 828
**Portugal**	EUR	419 (279.9–598.5)	36 734	24 663	20 852	20 852	15 391 422	10 333 680	8 737 064	8 737 064
**Romania**	RON	185.6 (125.9–258.5)	36 114	14 864	12 567	61 839	6 681 035	2 749 766	2 324 911	11 440 127
**Russia**	RUB	107.7 (76.2–148.4)	34 222	12 659	10 703	932 408	3 661 751	1 354 539	1 145 254	99 767 678
**San Marino**	EUR	247.5 (163–356.4)	67 831	51 809	43 805	43 805	16 754 356	12 796 916	10 819 715	10 819 715
**Serbia**	RSD	186.1 (140.9–242.9)	21 525	9180	7762	912 472	4 003 580	1 707 514	1 443 693	169 719 864
**Slovakia**	EUR	124.2 (84.4–174.4)	34 449	21 733	18 375	18 375	4 271 725	2 694 874	2 278 499	2 278 499
**Slovenia**	EUR	218.7 (144.6–313.2)	43 944	29 279	24 755	24 755	9 579 871	6 382 812	5 396 629	5 396 629
**Spain**	EUR	202.6 (142.5–278.8)	40 666	30 114	25 462	25 462	8 214 595	6 083 099	5 143 224	5 143 224
**Sweden**	SEK	316.8 (212.7–453.5)	60 323	61 358	51 878	526 244	19 062 098	19 389 233	16 393 480	166 293 052
**Switzerland**	CHF	238.3 (158.3–339.7)	76 251	92 343	78 075	84 387	18 147 637	21 977 593	18 581 922	20 084 136
**Tajikistan**	TJS	150.9 (109.8–198.8)	4382	917	775	10 367	657 306	137 504	116 259	1 555 005
**Türkiye**	TRY	244.7 (175.7–332)	30 452	9661	8169	85 506	7 430 312	2 357 340	1 993 116	20 863 416
**Turkmenistan**	TMT	115 (83.3–156)	17 219	10 150	8581	35 523	1 980 216	1 167 196	986 857	4 085 186
**Ukraine**	UAH	101.9 (65.7–147.2)	13 516	4596	3886	125 417	1 365 147	464 231	392 505	12 667 102
**United Kingdom**	GBP	429.9 (293.4–600.2)	49 818	46 410	39 240	33 743	21 371 750	19 909 987	16 833 774	14 475 855
**Uzbekistan**	UZS	213 (160.5–279.2)	8705	2031	1718	21 553 886	1 854 229	432 699	365 844	4 590 977 788
**WHO European Region**	NA	211.7 (148.1–288.8)	39 501	27 553	23 296	NA	8 334 703	5 813 713	4 915 459	NA

NCU: National Currency Unit; Int$: International Dollars; USD: United States Dollars; EUR: Euro; ALL: Albanian Lek; AMD: Armenian Dram; AZN: Azerbaijan Manat; BYN: Belarusian Ruble; BAM: Bosnia-Herzegovina Convertible Mark; BGN: Bulgarian Lev; CZK: Czech Koruna; DKK: Danish Krone; GEL: Georgian Lari; HUF: Hungarian Forint; ISK: Icelandic Krona; ILS: Israeli New Shekel; KZT: Kazakhstani Tenge; MKD: Macedonian Denar; MDL: Moldovan Leu; NOK: Norwegian Krone; PLN: Polish Złoty; RON: Romanian Leu; RUB: Russian Ruble; RSD: Serbian Dinar; SEK: Swedish Krona; CHF: Swiss Franc; TJS: Tajikistani Somoni; TRY: Turkish Lira; TMT: Turkmenistani Manat; UAH: Ukrainian Hryvnia; GBP: Great British Pound; UZS: Uzbekistani Som; NA: not applicable. **^#^**: an example of how to use these societal cost estimates is to state that WHO Europe should seek to invest USD 5 813 713 to eradicate the 211.7 (95% CI 148.1–288.8) DALY burden associated with asthma per 100 000 people (*i.e.* USD 58.14 per person). Other examples based on Austria and Türkiye are provided in the main article; ^¶^: data on GDP per capita are converted to a common currency (*i.e.* international dollars, Int$) using a purchasing power parity (PPP) adjustment (Int$2021). Relative to converting all currencies to a common currency using an exchange rate (*e.g.* USD), PPP-adjusted figures are more stable over time and allow a better comparison between countries; ^+^: although Greenland is reported separately to Denmark in table 2, our GDP per capita data source does not provide estimates for Greenland, thus Greenland is not reported separately here.

### GBD data covering nine lung conditions

Lung Facts provides GBD-reported epidemiological estimates for nine lung conditions, by age and sex, by and across all 53 WHO European countries and associated regions. Table 2 provides illustrative results for asthma.

### Non-GBD data covering five lung conditions

Data relating to conditions not in the GBD were systematically sought. A Preferred Reporting Items for Systematic Reviews and Meta-Analyses (PRISMA) flow diagram relating to the process of study selection is provided in supplementary file S2. In 2022, 1622 records were identified by the bibliographic and supplementary searches. Of these, 1493 were excluded based on their title or abstract. Full texts of 129 records were obtained, with data extracted from 16 studies: two cystic fibrosis reviews [15, 16] (one non-systematic) [16], one OSA systematic review [17], one influenza GBD study [18], one A1AD systematic review [19], six bronchiectasis primary studies [20–25], and five pulmonary arterial hypertension studies (two systematic reviews, three primary studies) [26–30].

In 2024, the updated search found 199 unique records; 172 were excluded based on their title or abstract. Full texts of 27 records were obtained, with 10 [31–40] included in the mapping review. 10 additional studies were identified through citation tracking; eight were excluded, with two [41, 42] included in the mapping review.

Figure 1 provides an evidence map of the data sourced for Lung Facts for non-GBD-included conditions. Supplementary file S2 shows a PRISMA flow diagram relating to the process of study selection and reasons for study exclusions are provided in supplementary file S3.

#### Cystic fibrosis

No data were identified for some countries across all outcomes, with no data broken down by age or sex. One systematic review [15] estimated prevalence across 40 European countries. One nonsystematic review [16] reported incidence-ratios across 19 European countries. One 2024 study provided Iceland [35], and another whole-Europe only [34], estimates. Overall, the systematic review data were considered more consistent and included in Lung Facts.

#### Obstructive sleep apnoea

No data were identified for incidence or mortality. One systematic review [17] modelled worldwide country-specific (50 European-specific) prevalence rates/numbers, based on 18 countries’ data (six European: Poland, Germany, Iceland, Norway, Spain and Switzerland) including by age and sex. Four prevalence-related systematic reviews [33, 36, 38, 40] were identified in 2024, but none were superior to the previous review [17] as they included fewer countries [33, 38, 40], reported fewer outcome data [33, 36, 40] or were in specific age groups only (one in children [38]; one in people aged ≥65 years [36]).

#### Alpha-1 antitrypsin deficiency

There were no data on incidence or mortality. One review [19] based on a systematic search calculated expected prevalence rates/numbers for 26 countries using Z-allele frequency and autosomal recessive inheritance. Four new studies [31, 32, 41, 42] were identified in 2024 covering Denmark, Germany, Madeira and La Palma; however, previous review data were retained to maintain consistency between countries.

#### Bronchiectasis

There were few data across countries and outcomes. Six primary studies [20–25] found in 2022 reported incidence, prevalence and mortality data for the United Kingdom, Poland, Germany, Italy and Spain. No new studies were found in 2024.

#### Influenza

One GBD study found in 2022, using data from 2017, attributed influenza to a fraction of the GBD's “lower respiratory tract infections” incidence and mortality estimates. One new study [37] found in 2024 was based on data older than 2017.

### Societal cost estimates

Table 3 provides illustrative asthma mean societal costs for 53 WHO Europe countries per 100 000 people. We focus on mean societal costs, with DALY 95% confidence intervals presented to indicate uncertainty impacting the societal cost estimates.

Based on two example countries chosen because they have quite different GDP per capita, but similar asthma DALY burden, ways to use these societal costs (USD) could be to state:

- Türkiye should aim to invest up to USD 2 357 340 to eradicate the 244.7 (175.7–332) DALY burden associated with asthma per 100 000 people (*i.e.* USD 23.57 per person);
- Austria should aim to invest up to USD 11 077 984 to eradicate the 206.0 (138.9–291.7) DALY burden associated with asthma per 100 000 people (*i.e.* USD 110.78 per person).

As these societal costs are presented as rates per 100 000 people, the main difference between countries is the DALY burden and GDP per capita. For example, while Austria has the lower asthma mean DALY burden rate compared to Türkiye (206.0 *versus* 244.7), Austria has the higher GDP per capita (USD 53 777 *versus* USD 9661), making the societal cost higher for Austria than Türkiye. This does not mean that Austria values avoiding the asthma burden more than Türkiye, rather it means that due to Austria's wealthier economy, the amount Austria could invest in lung care is estimated to be higher than the amount Türkiye could invest. In this context, the societal cost is how much a country could consider investing in lung care to tackle the condition burden within that society. Further ways to use these societal costs to inform policy-making are described by Franklin *et al.* [13].

## Discussion

Epidemiological estimates of respiratory health need regular review as determinants (*e.g.* exposure patterns, sociopolitical factors) change over time and geographies [43]. Accurate data are a critical component to developing national respiratory health plans. Lung Facts' methodology allows for relatively easy comparisons within and across a large number of WHO European countries, helping national coalitions assess and monitor policy progress over time. Lung Facts is largely populated with GBD estimates, to facilitate replicability and regular updates.

### Current evidence gaps

The GBD methodology is becoming the gold standard in population health data [44], used by most major administrations and organisations. Where GBD included the condition, estimates were complete across countries, estimates, ages and sexes. However, for non-GBD-included conditions, there were data gaps.

A1AD has particularly poor coverage, with prevalence based on allele frequency rather than diagnoses, though the 2024 update did identify some data on diagnoses in Germany [42] and Denmark [31]. Bronchiectasis has significant data gaps, with only six primary studies covering five countries and no new studies found in 2024 [20–25]. For cystic fibrosis and OSA, reviews identified cross-country epidemiological estimates using data from multiple sources, but not for mortality (both conditions) or incidence (OSA). The studies used different methodologies; thus, estimates may not be interchangeable nor comparable with GBD estimates. GBD's influenza study, based on 2017 lower respiratory infection data, did not report prevalence, YLL nor DALYs; no update was identified and influenza is not a current GBD condition.

### Filling evidence gaps

The GBD collates and analyses a huge amount of data to produce its global estimates. Harnessing GBD's high-quality methodology and expertise would be efficient to provide reasonably consistent estimation across countries and conditions. While not as comprehensive as the GBD, our research has filled evidence-gaps for some conditions (cystic fibrosis, OSA) while highlighting gaps for others (A1AD, bronchiectasis). Also, increasing GBD's current four-level condition categorisation could support more granular condition-specific estimation, *e.g.* influenza within lower respiratory infections, sarcoidosis within interstitial lung disease.

There are a few examples of national and international initiatives to map and monitor the epidemiology of respiratory conditions (European Community Respiratory Health Survey, www.ecrhs.org/; Respiratory Health in Northern Europe, https://breathesweden.com/rhine; the International Study of Asthma and Allergies in Childhood (ISAAC), https://isaac.auckland.ac.nz) [45, 46, 47]. For example, ISAAC [47] is a project investigating variations in the prevalence of asthma rhinitis and eczema at the population level, and their potential causes. The largest worldwide collaborative research project ever undertaken in children, it involves 306 centres in 105 countries, with nearly 2 million children participating and >1240 PubMed-indexed publications. Similar initiatives could be replicated across other respiratory conditions where data are sparse.

### Collated data: strengths and limitations

Our evidence review has helped populate Lung Facts, providing policy-makers and clinicians with a one-stop resource containing important statistics to help advocate for improved respiratory health. Lung Facts has already supported care and decision-making, *e.g.* the Belgian Lung Foundation used Lung Facts to emphasise the need to improve COPD patient care, and set strong objectives focusing on advocacy, better prevention and improved rehabilitation access.

Limitations of our approach include the focussed search strategies: while efforts were taken to identify all relevant data (*i.e.* citation searching, reference checking, asking experts), some studies may have been missed. It is well-known that bibliographic searches are generally only ∼80% [6] sensitive. Our additional search techniques identified 37.5% of the studies included in 2022 (an excess over comprehensive searches of 17.5%), and 17% in the 2024 update. However, only systematic reviews were included for some conditions, thus primary studies published since the most recent systematic review will have been missed.

### Societal costs: strengths and limitations

Our GDP-based approach for generating societal cost estimates is based on theory because there are no defined or empirically-based monetary DALY values, nor sufficient direct and indirect lung health cost estimates, for all WHO European countries. Despite this, decision-makers regularly need to prioritise resource allocation within countries, across conditions and associated population groups. As such, understanding the monetary cost of lung conditions is vital, but evidence is lacking.

The strengths and limitations of this GDP-based approach are described in detail elsewhere [13]. A key point is that monetised DALYs based on GDP are by intention and design a simplistic approach to valuing DALYs. The simplicity of the approach also aids its usefulness, if decision-makers are willing to utilise the valuation as a reference point [13]. For example, Drake [48] suggests a monetary DALY value, even with limitations and flaws, could be justified as it could “radically improve transparency and efficiency of priority setting in global health”, providing examples including the Millennium Development Goals and absolute poverty threshold.

Internationally, monetary valuations of the QALY for health technology assessment cost-effectiveness thresholds are also not evidence-based; rather they are decided by decision-makers as “approval norms” to support and rationalise resource allocation and care price negotiation within a fixed budget [13, 49]. The logic and necessity are similar to the GDP-based approach, but our DALY valuation is based on an economic activity metric (GDP per capita) rather than the QALY's “expert opinion” valuation [13].

The monetised DALY figures in Lung Facts do not replace the need for rigorously estimated economic data (*e.g.* understanding the care costs for conditions), but the benchmarked figures can be used as a reference point for investment to alleviate condition burden, even if the underlying model for monetising DALYs has flaws [13].

### Conclusion

There are evidence gaps for several respiratory conditions that have an epidemiological and economic burden on people, their families/carers, and society. Researchers, including the GBD study, should extend their scope to additional respiratory conditions, including A1AD, cystic fibrosis, OSA and bronchiectasis. New evidence is needed to benefit more people burdened by poor lung health internationally. Lung Facts provides epidemiological and economic data for WHO European countries for a range of lung conditions, to support clinicians and policy-makers in advocating for lung health and in setting up respiratory plans.

## Supplementary material

**Please note:** supplementary material is not edited by the Editorial Office, and is uploaded as it has been supplied by the author. Search strategoes and data extraction 01351-2025.SUPPLEMENT PRISMA flow diagram 01351-2025.SUPPLEMENT2 Studies excluded from both reviews 01351-2025.SUPPLEMENT3
