# Supplementary material for: The epidemiological burden and societal cost of 14 respiratory conditions in the World Health Organization European region: systematic evidence map and economic analysis
Source: ERJ Open Res. 2026 Jun 29;12(3):01351-2025. doi: 10.1183/23120541.01351-2025 (PMC13312043; doi:10.1183/23120541.01351-2025)
Supplement: Supplementary file 1 [file 01351-2025.SUPPLEMENT.pdf]

Online Supplementary File: Search strategies and data extraction

This document contains:

1. Search strategy from 2024
2. Search strategy from 2022
3. Data extraction fields

1. Search strategy from 2024

An update search was run in May 2024, based on the original search conducted in May 2022. Two separate searches were conducted; 1) to identify systematic reviews on cystic fibrosis, sleep apnoea, and influenza, 2) Alpha-1 and Bronchiectasis with no limits on study design. Searches were conducted on MEDLINE, Embase, and the Cochrane Library, and limited to studies published since May 2022. The full search strategies and the number of records found in each database can be found below. Experts were also consulted for any missed studies.

Search 1: Systematic Reviews - Cystic Fibrosis, Sleep Apnoea, Influenza

**Database**	**Date Search**	**No. of records retrieved**
Ovid MEDLINE(R) Epub Ahead of Print and In-Process, In-Data-Review & Other Non-Indexed Citations and Daily <May 21, 2024>	22/05/24	59
Embase <1974 to 2024 Week 20>	22/05/24	106
Cochrane Database of Systematic Reviews<br>Issue 5 of 12, May 2024	22/05/24	6
Total		171
**Total after duplicates removed**		**125**

Search 1 Search Strategies

Ovid MEDLINE(R) Epub Ahead of Print and In-Process, In-Data-Review & Other Non-Indexed Citations and Daily <May 21, 2024>

1 cystic fibrosis.ti. 32758

2 sleep apn?ea*.ti. 29743

3 osahs.ti. 216

4 (influenza* or flu or grippe).ti. 95574

5 or/1-4 158151

6 Epidemiologic Studies/ 9546

7 (epidemiology or epidemiological).ti.120322

8 Incidence/ or incidence.ti. 356530

9 Prevalence/ or prevalence.ti. 412622

10 Disability-Adjusted Life Years/ 292

11 disability adjusted life.mp. 6206

12 (DALY or DALYS).mp. 5440

13 "years of life lost".mp. 2611

14 YLL.ti.19

15 Mortality/ 49936

16 mortality.ti. 167904

17 or/6-161021634

18 5 and 17 9975

19 meta analysis.mp,pt. or review.pt. or search:.tw. 3848319

20 18 and 19 1150

21 ("20220528" or "20220529" or "20220530" or "20220531").dt. 17216

22 (2023* or 2024*).dt. 2198413

23 21 or 22 2215629

24 20 and 23 59

Embase <1974 to 2024 Week 20>

1 cystic fibrosis.ti. 49534

2 sleep apn?ea*.ti. 45377

3 osahs.ti. 306

4 (influenza* or flu or grippe).ti. 101561

5 or/1-4 196544

6 *epidemiology/ 48559

7 (epidemiology or epidemiological).ti. 140150

8 *incidence/ 40577

9 incidence.ti. 155850

10 *prevalence/ 105512

11 prevalence.ti. 237746

12 *disability-adjusted life year/ 618

13 disability adjusted life.mp. 9232

14 (DALY or DALYS).mp. 7030

15 "years of life lost".mp. 3284

16 YLL.ti.20

17 *mortality/ 118224

18 mortality.ti. 217917

19 or/6-18814671

20 (2022* or 2023* or 2024*).dc. 5182087

21 5 and 19 and 20 1165

22 (((systematic or state-of-the-art or scoping or literature or umbrella) adj (review* or overview* or assessment*)) or "review* of reviews" or meta-analy* or metaanaly* or ((systematic or evidence) adj1 assess*) or "research evidence" or metasynthe* or meta-synthe*).tw. or systematic review/ or "systematic review (topic)"/ or meta analysis/ or "meta analysis (topic)"/ 962200

23 21 and 22 106

Cochrane Library (CDSR & CENTRAL)

#1 MeSH descriptor: [Epidemiologic Studies] this term only 129

#2 (epidemiology or epidemiological):ti 1107

#3 MeSH descriptor: [Incidence] this term only 14333

#4 incidence:ti 8616

#5 MeSH descriptor: [Prevalence] this term only 7402

#6 prevalence:ti 3478

#7 MeSH descriptor: [Disability-Adjusted Life Years] explode all trees 4

#8 disability adjusted life 2822

#9 (DALY or DALYS) 1628

#10 "years of life lost" 67

#11 YLL:ti 0

#12 MeSH descriptor: [Mortality] this term only 1029

#13 mortality:ti 7809

#14 1-#13 41877

#15 cystic fibrosis:ti 4857

#16 sleep apn?ea*:ti 5540

#17 osahs:ti 57

#18 (influenza* or flu or grippe):ti 6112

#19 #15 or #16 or #17 or #18 16539

#20 #14 and #19 with Cochrane Library publication date Between Jan 2022 and May 2024 23

Search 1: Alpha-1, Bronchiectasis

**Database**	**Date Search**	**No. of records retrieved**
Ovid MEDLINE(R) Epub Ahead of Print and In-Process, In-Data-Review & Other Non-Indexed Citations and Daily <May 17, 2024>	20/05/24	26
Embase <1974 to 2024 Week 20>	20/05/24	78

Cochrane Database of Systematic Reviews<br>Issue 5 of 12, May 2024	20/05/24	1
Cochrane Central Register of Controlled Trials<br>Issue 4 of 12, April 2024	20/05/24	1
Total		106
**Total after duplicates removed**		**74**

Ovid MEDLINE(R) Epub Ahead of Print and In-Process, In-Data-Review & Other Non-Indexed Citations and Daily <May 17, 2024>

1 bronchiectas*.ti. 5321

2 ((alpha 1 or alpha-1) adj antitrypsin deficienc*).ti. 1890

3 Epidemiologic Studies/ 9545

4 (epidemiology or epidemiological).ti.120261

5 Incidence/ or incidence.ti. 356376

6 Prevalence/ or prevalence.ti. 412427

7 Disability-Adjusted Life Years/ 281

8 disability adjusted life.mp. 6191

9 (DALY or DALYS).mp. [mp=title, book title, abstract, original title, name of substance word, subject heading word, floating sub-heading word, keyword heading word, organism supplementary concept word, protocol supplementary concept word, rare disease supplementary concept word, unique identifier, synonyms, population supplementary concept word, anatomy supplementary concept word] 5428

10 "years of life lost".mp. 2607

11 YLL.ti.19

12 Mortality/ 49915

13 mortality.ti. 167753

14 1 or 2 7185

15 or/2-13 1021095

16	14 and 15	390
17	("20220528" or "20220529" or "20220530" or "20220531").dt.	17216
18	(2023* or 2024*).dt.	2181707
19	17 or 18	2198923
20	16 and 19	26

Embase <1974 to 2024 Week 20>

1	bronchiectas*.ti.	6274
2	((alpha 1 or alpha-1) adj antitrypsin deficienc*).ti.	2129
3	*epidemiology/	48559
4	(epidemiology or epidemiological).ti.	140150
5	*incidence/	40577
6	incidence.ti.	155850
7	*prevalence/	105512
8	prevalence.ti.	237746
9	*disability-adjusted life year/	618
10	disability adjusted life.mp.	9232
11	(DALY or DALYS).mp.	7030
12	"years of life lost".mp.	3284
13	YLL.ti.	20
14	*mortality/	118224
15	mortality.ti.	217917
16	1 or 2	8370
17	or/3-15	814671
18	16 and 17	349
19	(2022* or 2023* or 2024*).dc.	5182087
20	18 and 19	78

Cochrane Library (CDSR & CENTRAL)

#1	(bronchiectas*):ti	923
#2	((alpha 1 or alpha-1) NEAR/1 antitrypsin deficienc*):ti	74
#3	MeSH descriptor: [Epidemiologic Studies] this term only	129
#4	(epidemiology or epidemiological):ti	1107
#5	MeSH descriptor: [Incidence] this term only	14333
#6	incidence:ti	8616
#7	MeSH descriptor: [Prevalence] this term only	7402
#8	prevalence:ti	3478
#9	MeSH descriptor: [Disability-Adjusted Life Years] explode all trees	4
#10	disability adjusted life	2821
#11	(DALY or DALYS)	1628
#12	"years of life lost"	67
#13	YLL:ti	0
#14	MeSH descriptor: [Mortality] this term only	1029
#15	mortality:ti	7809
#16	#1 OR #2	997
#17	{OR #3-#15}[2]	41876
#18	#16 and #17	13
#19	#18 with Cochrane Library publication date Between Jan 2022 and May 2024	2

2. Search strategy from 2022

A search of Medline was conducted in May 2022. A focussed approach to searching was taken, and only one database was searched due to the large number of records that would be retrieved were more exhaustive methods used (in excess of 15,000 records) and budgetary considerations. Because Medline has been estimated to identify approximately 85% of all records in systematic reviews,[2] this was selected as the database to search. Additional search techniques were also applied, which included contact with experts, via ELF, and snowballing (checking citations in Google Scholar, and checking reference lists) from important studies,

where insufficient data were identified by the database search. For two conditions (influenza and cystic fibrosis), additional focussed searches were conducted in Google Scholar, since no relevant studies were identified by the initial Medline search.

Ovid MEDLINE(R) and Epub Ahead of Print, In-Process, In-Data-Review & Other Non-Indexed Citations and Daily <1946 to May 26, 2022>

1 bronchiectas*.ti. 4836

2 cystic fibrosis.ti. 30612

3 sleep apn?ea*.ti. 26352

4 osahs.ti. 196

5 (pulmonary hypertension or pulmonary arterial hypertension or chronic thromboembolic pulmonary hypertension or CTEPH).ti. 27597

6 ((alpha 1 or alpha-1) adj antitrypsin deficienc*).ti. 1763

7 Epidemiologic Studies/ 9129

8 (epidemiology or epidemiological).ti.110794

9 Incidence/ or incidence.ti. 340250

10 Prevalence/ or prevalence.ti. 385440

11 Disability-Adjusted Life Years/ 126

12 disability adjusted life.mp. 4551

13 (DALY or DALYS).mp. [mp=title, abstract, original title, name of substance word, subject heading word, floating sub-heading word, keyword heading word, organism supplementary concept word, protocol supplementary concept word, rare disease supplementary concept word, unique identifier, synonyms] 3973

14 "years of life lost".mp. 2125

15 YLL.ti.12

16 Mortality/ 48911

17 mortality.ti. 147292

18 meta analysis.mp,pt. or review.pt. or search:.tw. 3449004

19 or/7-17949712

20 2 or 3 or 4 57123

21	19 and 20	3685
22	18 and 21	603
23	limit 22 to yr="2010 -Current"	382
24	1 or 5 or 6	34168
25	19 and 24	1566
26	limit 25 to yr="2010 -Current"	**1130 All studies (unfiltered) since 2010 for bronchiectas, alpha 1, PH**
27	(influenza* or flu or grippe).ti.	89426
28	10 or 11 or 12 or 13 or 14 or 15	391442
29	18 and 27 and 28	90
30	limit 29 to yr="2010 -Current"	69
31	23 or 30	**448 Reviews since 2010 for CF, apnoea, influenza***

***prevalance, DALYs and YLL only for influenza**

3. Data extraction fields

Data were extracted under the following headings:

Author, year

Study design (systematic review (SR) or primary; retrospective, prospective)

Source of extracted data (if primary study extracted from systematic review)

Year of data collection

Condition

Country

Sex & Age inclusion criteria

SR search date

Source of primary study data (registry, health insurance claims etc)

Incidence rates, incidence numbers

Prevalence rates, prevalence numbers

Mortality rates, mortality numbers

DALY rates, DALY numbers

YLL rates, YLL numbers

Notes

1. Netikul T, Palittapongarnpim P, Thawornwattana Y, et al. Estimation of the global burden of Mycobacterium tuberculosis lineage 1. *Infection, Genetics & Evolution* 2021;91:104802.
2. Booth A. Over 85% of included studies in systematic reviews are on MEDLINE. *J Clin Epidemiol* 2016;79:165-66. doi: 10.1016/j.jclinepi.2016.04.002 [published Online First: 20160421]
