# Supplementary material for: The epidemiological burden and societal cost of 14 respiratory conditions in the World Health Organization European region: systematic evidence map and economic analysis
Source: ERJ Open Res. 2026 Jun 29;12(3):01351-2025. doi: 10.1183/23120541.01351-2025 (PMC13312043; doi:10.1183/23120541.01351-2025)
Supplement: Supplementary file 2 [file 01351-2025.SUPPLEMENT2.pdf]

Online Supplementary File: PRISMA flow diagram

It should be noted that there is a difference in the number of records included in 2022 (n=16) and the number of studies included in previous versions of the review in 2024 (n=11). This is because PAH data was sourced from the GBD in 2024 when it was added as a new category, and the 5 studies on PAH from 2022 were therefore not required in 2024.

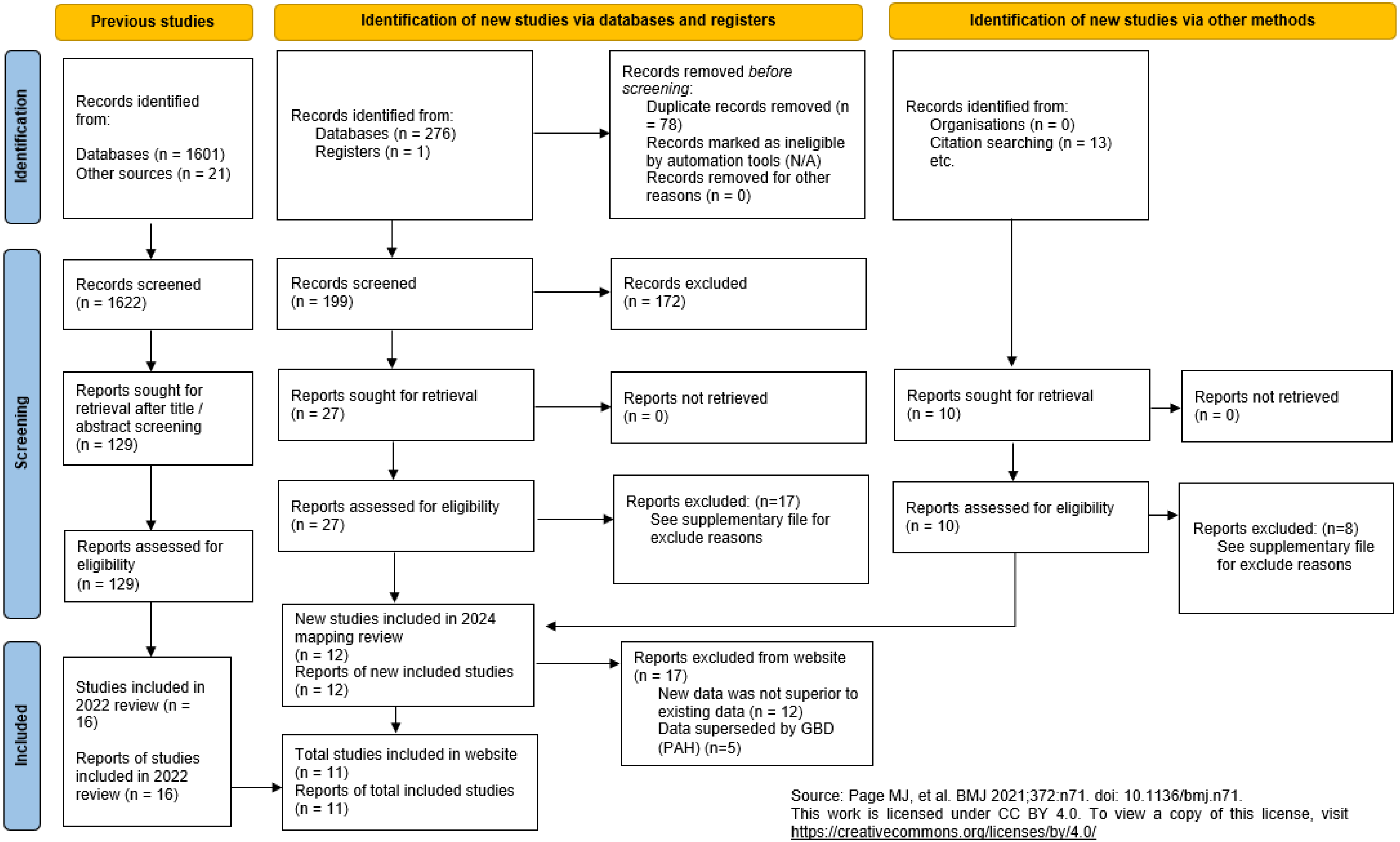

Source: Page MJ, et al. BMJ 2021;372:n71. doi: 10.1136/bmj.n71.
This work is licensed under CC BY 4.0. To view a copy of this license, visit https://creativecommons.org/licenses/by/4.0/
