# Supplementary material for: The epidemiological burden and societal cost of 14 respiratory conditions in the World Health Organization European region: systematic evidence map and economic analysis
Source: ERJ Open Res. 2026 Jun 29;12(3):01351-2025. doi: 10.1183/23120541.01351-2025 (PMC13312043; doi:10.1183/23120541.01351-2025)
Supplement: Supplementary file 3 [file 01351-2025.SUPPLEMENT3.pdf]

Online Supplementary File S3: Studies excluded from both reviews

This supplement contains a list of studies excluded from the 2022 review, for each condition as follows:

- Table 1 Studies excluded from the 2022 review of cystic fibrosis (n=4)
- Table 2 Studies excluded from the 2022 review of obstructive sleep apnoea (n=17)
- Table 3 Studies excluded from the 2022 review of influenza (n=9)
- Table 4 Studies excluded from the 2022 review of Alpha-1 (n=14)
- Table 5 Studies excluded from the 2022 review of Bronchiectasis (n=21)
- Table 6 Studies excluded from the 2022 review of PAH (n=48)

Further lists are provided of studies excluded from the 2024 review as follows:

- Table 7 Studies excluded from the 2024 review (bibliographic databases) (n=17)
- Table 8 Studies excluded from the 2024 review (other sources) (n=8)

Table 1 Studies excluded from the 2022 review of cystic fibrosis

Author, year	Exclude reason
Parkins 2011[1]	Exclude - not SR, no data on any outcomes
Salvatore 2017[2]	Exclude - SR, but no outcomes of interest
Scotet 2020a[3]	Exclude – refers to another review, Scotet 2020b, which is listed above
Spoonhower 2016[4]	Exclude - not SR

Table 2 Studies excluded from the 2022 review of obstructive sleep apnoea

Author, year	Exclude reason
Barewal 2019[5]	Not an SR
Dudley 2016[6]	No outcomes of interest (risk factor study)
Franklin 2013[7]	Primary study, but check if included in srs? (not included in Benjafield 2019)
Franklin 2015[8]	Not an SR

Fu 2017[9]	SR of multivariable adjusted hazard ratios for all-cause mortality
Garvey 2015[10]	Not an SR
Ge 2013[11]	SR of multivariable adjusted hazard ratios for all-cause mortality
Maspero 2015[12]	Not a high quality SR, only includes one study in Europe (Italy)
Pan 2016[13]	SR of mortality hazard ratios
Peppard 2018[14]	Not an SR, expert review
Sarkar 2018[15]	OSA and cardiovascular outcomes/comorbidities
Senaratna 2017[16]	Does not report per country, superseded by Benjafield
Theorell-Haglow 2018[17]	Not an SR
Trzepizur 2014[18]	Not an SR
Turanyi 2014[19]	Not an SR
Vlaipour 2012[20]	Not an SR
Wimms 2016[21]	Not an SR

Table 3 Studies excluded from the 2022 review of influenza

**Author, year**	**Exclude reason**
Furuya-Kanamori 2016[22]	SR of asymptomatic flu only
Huang 2022[23]	Only reports loss of life expectancy, not YLL
Luliano 2018[24]	Influenza-related respiratory mortality, 2015; superseded by GBD 2017 influenza collaborators
Lafond 2021[25]	SR of adult flu, 2016 data – superseded by GBD 2017 influenza collaborators
Nair 2011[26]	SR to 2010, too old
Nielsen 2019[27]	Data only for Europe as a whole
Paget 2022[28]	Old data, 2011, superseded by by GBD 2017 influenza collaborators
Wang 2020[29]	Paediatric data only

Wang 2022[30]	Paediatric data only

Table 4 Studies excluded from the 2022 review of Alpha-1

Author, year	Exclude reason
Blanco 2017a[31]	Only pisz/pis/piz
Blanco 2018[32]	Letter to the editor relating to study in the 1990's
Blanco 2020[33]	Only in patients with COPD
Carroll 2011[34]	In SR
Chorostowska-Wynimko 2012[35]	In SR
de Serres 2014[36]	Superseded by more recent publications
Haggblom 2015[37]	Pre 2010 data (2000-2001)
Hutsebaut 2015[38]	Expert review - source of earlier studies? (2 from belgium, 1990/91)
Karl 2017[39]	Only in patients with COPD
Kelly 2010[40]	Expert review; pre-2010 data (1986; 1994)
Martinez-González 2021[41]	Only pimz
Miravitlles 2022[42]	Does not report any relevant outcomes
Mostafavi 2019[43]	Survival expressed as standardised mortality ratio
Poplawska 2013[44]	No data on outcomes of interest

Table 5 Studies excluded from the 2022 review of Bronchiectasis

Author, year	Exclude reason
Chandrasekaran 2018[45]	Poor quality SR – used as a source for studies only
Diel 2019[46]	Superseded by Ringhausen 2019
Monteagudo 2016[47]	Sanchez-Munoz 2016[48] is national data
Navarro-Rolon 2021[49]	No outcome of interest - annual rate of admissions is not a proxy for incidence or prevalence

Ringshausen 2015[50]	Superseded by Ringhausen 2019
Snell 2019[51]	Superseded by Quint 2016[52] and Gayle 2019[53]
Bellelli 2016[54]	No data on outcomes of interest (recruited PH patients only)
Gao 2016[55]	No relevant outcomes
Masters 2010[56]	Conference abstract; pre-2010 data; data not in correct format (per general practitioner)
Navaratnam 2017[57]	No outcomes of interest (incidence of stroke/CHD in Bronchiectasis patients)
O'Donnell 2018[58]	Expert review
Polverino 2012[59]	Conference abstract with not enough detail to use, though there are some weighted mean incidence estimates for EU big 5
Quint 2019[60]	Expert review, no new references
Roberts 2010[61]	Data pre-2010
Anwar 2013[62]	No data on outcomes of interest (recruited PH patients only)
Dimakou 2016[63]	No data on outcomes of interest (recruited PH patients only)
Buscot 2016[64]	No data on outcomes of interest (recruited PH patients only)
Lonni 2015[65]	No data on outcomes of interest (recruited PH patients only)
Martinez-Garcia 2017[66]	No data on outcomes of interest (recruited PH patients only)
Saynajakangas 1997[67]	Data from pre 2010
Saynajakangas 1998[68]	Data from pre 2010

Table 6 Studies excluded from the 2022 review of PAH

Author, year	Exclude reason
Auger 2010[69]	Data pre 2010
Condliffe 2011[70]	Data pre 2010
Escribano-Subias 2012[71]	Data pre 2010
Fraisse 2010[72]	Data pre 2010
Ling 2012[73]	Data pre 2010

van Loon 2011[74]	Data pre 2010
Delcroix 2016[75]	Expert review
Fernandes 2018[76]	Expert review
Franco 2019[77]	Expert review
Jiang 2013[78]	Expert review; mostly pre 2010 data
Jin 2012[79]	Expert review; only children
Klok 2010[80]	Expert review; pre-2010 data
Lau 2017[81]	Expert review
Levine 2021[82]	Expert review
McGoon 2013[83]	Expert review; mostly pre 2010 data
Medrek 2016[84]	Expert review
Mocumbi 2015[85]	Expert review
Orem 2017[86]	Expert review in the elderly
Prins 2016[87]	Expert review
Swinnen 2019[88]	Expert review
Taichman 2013[89]	Expert review
Thenappan 2012[90]	Expert review; mostly pre 2010
Clark 2016[91]	Study design recruits too narrow population (Group 2 PH only)
Coquoz 2018[92]	Study design recruits too narrow population (PE patients)
Georgiopoulou 2013[93]	Study design recruits too narrow population (PH in heart failure)
Gerges 2015[94]	Study design recruits too narrow population (PH in heart failure)
Guha 2016[95]	Study design recruits too narrow population (PH in heart failure)
Thenappan 2014[96]	Study design recruits too narrow population
Cottin 2019[97]	Included in Leber 2021
Hoeper 2016[98]	Included in Leber 2021

Kramm 2018[99]	Included in Leber 2021
Pektas 2016[100]	Included in Leber 2021
Jansa 2014[101]	Superseded by Jansa 2022; pre 2010 data
Radegran 2016[102]	Later data in Leber 2021
Skride 2016[103]	Later data in Leber 2021
Arvanitaki 2019[104]	No data on outcomes of interest; study design recruits only PAH patients
Awdish 2016[105]	No data on outcomes of interest; study design recruits only PAH patients
Bergot 2019[106]	No data on outcomes of interest
Demerouti 2021[107]	No data on outcomes of interest
Dzudie 2014[108]	No data on outcomes of interest; study design recruits only subset of PH pts with left ventricular HF
Hoeper 2017[109]	No data on outcomes of interest
Humbert 2010[110]	No data on outcomes of interest; pre 2010 data
Imbalzano 2021[111]	No data on outcomes of interest: prevalence of intermediate or high risk of PAH, not actually diagnosed with PAH
Natalia 2020[112]	no data on outcomes of interest; prescription data from one hospital not adequate way to measure incidence or prevalence
Pepke-Zaba 2011[113]	No data on outcomes of interest; data collected pre-2010
Pfeuffer-Jovic 2021[114]	No data on outcomes of interest
Sinan 2019[115]	No data on outcomes of interest
Emmons-Bell 2022[116]	Systematic review, but no novel studies compared to Gall 2017 and Leber 2021

Table 7 Studies excluded from the 2024 review (bibliographic databases)

Chang 2022[117]	Not a systematic review
Krishnan 2023[118]	Not a systematic review

Sezgin 2023[119]	No new data
Karki 2022[120]	Insufficient data
Maggi 2022[121]	No data on Europe or flu separately
Kim 2023[122]	Not Europe
Soudani 2022[123]	No relevant data on Europe
Li 2021[124]	Hospital-acquired flu only
Polverino 2024[125]	no relevant data.
Landry 2023[126]	Only a subset of COPD
Betts 2022[127]	Not Europe
Abavisani 2024[128]	Not flu
Munteanu 2022[129]	No relevant data
Alzahrani 2023[130]	Not Europe
Stockley 2023[131]	Study design not at population level
Rezaei-Tavabe 2022[132]	Study design not at population level
Prechaporn 2024[133]	Study design not at population level

Table 8 Studies excluded from the 2024 review (other sources)

Senaratna 2017[16]	Excluded in 2022
Quint 2016[52]	Already included in 2022
Heinzer 2015[134]	Already included in Benjafield review (extracted in 2022)
Lonni 2015[135]	No relevant data
Martínez-Garcia 2018[136]	No data on prevalance
Chalmers 2023[137]	New, but unclear if SR for prevalence, refs 3 papers (Added to list), all were in 2022 searches. Have contacted co-author about Bronchiectasis registry.
Ringshausen 2019[138]	Was considered in 2022
Ringshausen 2015[50]	Was considered in 2022

1. Parkins MD, Parkins VM, Rendall JC, et al. Changing epidemiology and clinical issues arising in an ageing cystic fibrosis population. *Therapeutic Advances in Respiratory Disease* 2011;5(2):105-19.
2. Salvatore D, Buzzetti R, Mastella G. Update of literature from cystic fibrosis registries 2012-2015. Part 6: Epidemiology, nutrition and complications. *Pediatric Pulmonology* 2017;52(3):390-98.
3. Scotet V, Gutierrez H, Farrell PM. Newborn screening for CF across the globe—where is it worthwhile? *International journal of neonatal screening* 2020b;6(1):18.
4. Spoonhower KA, Davis PB. Epidemiology of Cystic Fibrosis. *Clinics in Chest Medicine* 2016;37(1):1-8.
5. Barewal RM. Obstructive Sleep Apnea: The Role of Gender in Prevalence, Symptoms, and Treatment Success. *Dental Clinics of North America* 2019;63(2):297-308.
6. Dudley KA, Patel SR. Disparities and genetic risk factors in obstructive sleep apnea. *Sleep Medicine* 2016;18:96-102.
7. Franklin KA, Sahlin C, Stenlund H, et al. Sleep apnoea is a common occurrence in females. *European Respiratory Journal* 2013;41(3):610-5.
8. Franklin KA, Lindberg E. Obstructive sleep apnea is a common disorder in the population-a review on the epidemiology of sleep apnea. *Journal of Thoracic Disease* 2015;7(8):1311-22.
9. Fu Y, Xia Y, Yi H, et al. Meta-analysis of all-cause and cardiovascular mortality in obstructive sleep apnea with or without continuous positive airway pressure treatment. *Sleep & Breathing* 2017;21(1):181-89.
10. Garvey JF, Pengo MF, Drakatos P, et al. Epidemiological aspects of obstructive sleep apnea. *Journal of Thoracic Disease* 2015;7(5):920-9.
11. Ge X, Han F, Huang Y, et al. Is obstructive sleep apnea associated with cardiovascular and all-cause mortality? *PLoS ONE [Electronic Resource]* 2013;8(7):e69432.
12. Maspero C, Giannini L, Galbiati G, et al. Obstructive sleep apnea syndrome: a literature review. *Minerva Stomatologica* 2015;64(2):97-109.
13. Pan L, Xie X, Liu D, et al. Obstructive sleep apnoea and risks of all-cause mortality: preliminary evidence from prospective cohort studies. *Sleep & Breathing* 2016;20(1):345-53.
14. Peppard PE, Hagen EW. The Last 25 Years of Obstructive Sleep Apnea Epidemiology-and the Next 25? *American Journal of Respiratory & Critical Care Medicine* 2018;197(3):310-12.
15. Sarkar P, Mukherjee S, Chai-Coetzer CL, et al. The epidemiology of obstructive sleep apnoea and cardiovascular disease. *Journal of Thoracic Disease* 2018;10(Suppl 34):S4189-S200.
16. Senaratna CV, Perret JL, Lodge CJ, et al. Prevalence of obstructive sleep apnea in the general population: A systematic review. *Sleep Medicine Reviews* 2017;34:70-81.
17. Theorell-Haglow J, Miller CB, Bartlett DJ, et al. Gender differences in obstructive sleep apnoea, insomnia and restless legs syndrome in adults - What do we know? A clinical update. *Sleep Medicine Reviews* 2018;38:28-38.
18. Trzepizur W, Gagnadoux F. [Epidemiology of obstructive sleep apnoea syndrome]. *Revue des Maladies Respiratoires* 2014;31(6):568-77.
19. Turanyi CZ, Pinter N, Dunai A, et al. [Obstructive sleep apnea in women]. *Orvosi Hetilap* 2014;155(52):2067-73.
20. Valipour A. Gender-related differences in the obstructive sleep apnea syndrome. *Pneumologie* 2012;66(10):584-8.

21. Wimms A, Woehrle H, Ketheeswaran S, et al. Obstructive Sleep Apnea in Women: Specific Issues and Interventions. *BioMed Research International* 2016;2016:1764837.
22. Furuya-Kanamori L, Cox M, Milinovich GJ, et al. Heterogeneous and Dynamic Prevalence of Asymptomatic Influenza Virus Infections. *Emerging Infectious Diseases* 2016;22(6):1052-6.
23. Huang G, Guo F. Loss of life expectancy due to respiratory infectious diseases: findings from the global burden of disease study in 195 countries and territories 1990–2017. *Journal of Population Research* 2022;39(1):1-43.
24. Iuliano AD, Roguski KM, Chang HH, et al. Estimates of global seasonal influenza-associated respiratory mortality: a modelling study. *The Lancet* 2018;391(10127):1285-300.
25. Lafond KE, Porter RM, Whaley MJ, et al. Global burden of influenza-associated lower respiratory tract infections and hospitalizations among adults: A systematic review and meta-analysis. *PLoS medicine* 2021;18(3):e1003550.
26. Nair H, Brooks WA, Katz M, et al. Global burden of respiratory infections due to seasonal influenza in young children: a systematic review and meta-analysis. *Lancet* 2011;378(9807):1917-30.
27. Nielsen J, Vestergaard LS, Richter L, et al. European all-cause excess and influenza-attributable mortality in the 2017/18 season: should the burden of influenza B be reconsidered? *Clinical microbiology and infection* 2019;25(10):1266-76.
28. Paget J, Iuliano AD, Taylor RJ, et al. Estimates of mortality associated with seasonal influenza for the European Union from the GLaMOR project. *Vaccine* 2022;40(9):1361-69.
29. Wang X, Li Y, O'Brien KL, et al. Global burden of respiratory infections associated with seasonal influenza in children under 5 years in 2018: a systematic review and modelling study. *The Lancet Global Health* 2020;8(4):e497-e510.
30. Wang X, Li Y, Mei X, et al. Global hospital admissions and in-hospital mortality associated with all-cause and virus-specific acute lower respiratory infections in children and adolescents aged 5–19 years between 1995 and 2019: a systematic review and modelling study. *BMJ global health* 2021;6(7):e006014.
31. Blanco I, Bueno P, Diego I, et al. Alpha-1 antitrypsin Pi SZ genotype: estimated prevalence and number of SZ subjects worldwide. *International Journal of Copd* 2017a;12:1683-94.
32. Blanco I. A well-designed/conducted study on alpha-1 antitrypsin epidemiology not quoted. *European Respiratory Journal* 2018;51(3):03.
33. Blanco I, Diego I, Bueno P, et al. Prevalence of α1-antitrypsin PiZZ genotypes in patients with COPD in Europe: a systematic review. *European Respiratory Review* 2020;29(157)
34. Carroll TP, O'Connor CA, Floyd O, et al. The prevalence of alpha-1 antitrypsin deficiency in Ireland. *Respiratory Research* 2011;12:91.
35. Chorostowska-Wynimko J, Struniawski R, Poplawska B, et al. [The incidence of alpha-1-antitrypsin (A1AT) deficiency alleles in population of Central Poland--preliminary results from newborn screening]. *Pneumonologia i Alergologia Polska* 2012;80(5):450-3.
36. de Serres F, Blanco I. Role of alpha-1 antitrypsin in human health and disease. *Journal of Internal Medicine* 2014;276(4):311-35.
37. Haggblom J, Kettunen K, Karjalainen J, et al. Prevalence of PI Z and PI S alleles of alpha-1-antitrypsin deficiency in Finland. *European Clinical Respiratory Journal* 2015;2:28829.

38. Hutsebaut J, Janssens W, Louis R, et al. Activity of the alpha-1 antitrypsin deficiency registry in Belgium. *Copd: Journal of Chronic Obstructive Pulmonary Disease* 2015;12 Suppl 1:10-4.
39. Karl FM, Holle R, Bals R, et al. Costs and health-related quality of life in Alpha-1-Antitrypsin Deficient COPD patients. *Respiratory Research* 2017;18(1):60.
40. Kelly E, Greene CM, Carroll TP, et al. Alpha-1 antitrypsin deficiency. *Respiratory Medicine* 2010;104(6):763-72.
41. Martinez-Gonzalez C, Blanco I, Diego I, et al. Estimated Prevalence and Number of PiMZ Genotypes of Alpha-1 Antitrypsin in Seventy-Four Countries Worldwide. *International Journal of Copd* 2021;16:2617-30.
42. Miravitlles M, Herepath M, Priyendu A, et al. Disease burden associated with alpha-1 antitrypsin deficiency: systematic and structured literature reviews. *European Respiratory Review* 2022;31(163)
43. Mostafavi B, Piitulainen E, Tanash HA. Survival in the Swedish cohort with alpha-1-antitrypsin deficiency, up to the age of 43-45 years. *International Journal of Copd* 2019;14:525-30.
44. Poplawska B, Janciauskiene S, Chorostowska-Wynimko J. [Genetic variants of alpha-1 antitrypsin: classification and clinical implications]. *Pneumonologia i Alergologia Polska* 2013;81(1):45-54.
45. Chandrasekaran R, Mac Aogain M, Chalmers JD, et al. Geographic variation in the aetiology, epidemiology and microbiology of bronchiectasis. *BMC Pulmonary Medicine* 2018;18(1):83.
46. Diel R, Ewig S, Blaas S, et al. Incidence of patients with non-cystic fibrosis bronchiectasis in Germany - A healthcare insurance claims data analysis. *Respiratory Medicine* 2019;151:121-27.
47. Monteagudo M, Rodriguez-Blanco T, Barrecheguren M, et al. Prevalence and incidence of bronchiectasis in Catalonia, Spain: A population-based study. *Respiratory Medicine* 2016;121:26-31.
48. Sanchez-Munoz G, Lopez de Andres A, Jimenez-Garcia R, et al. Time Trends in Hospital Admissions for Bronchiectasis: Analysis of the Spanish National Hospital Discharge Data (2004 to 2013). *PLoS ONE [Electronic Resource]* 2016;11(9):e0162282.
49. Navarro-Rolon A, Rosa-Carrillo Ddl, Esquinas C, et al. Evolution and Comparative Analysis of Hospitalizations in Spain Due to COPD and Bronchiectasis between 2004 and 2015. *COPD: Journal of Chronic Obstructive Pulmonary Disease* 2021;18(2):210-18.
50. Ringshausen FC, de Roux A, Diel R, et al. Bronchiectasis in Germany: a population-based estimation of disease prevalence. *European Respiratory Journal* 2015;46(6):1805-7.
51. Snell N, Gibson J, Jarrold I, et al. Epidemiology of bronchiectasis in the UK: Findings from the British lung foundation's 'Respiratory health of the nation' project. *Respiratory Medicine* 2019;158:21-23.
52. Quint JK, Millett ER, Joshi M, et al. Changes in the incidence, prevalence and mortality of bronchiectasis in the UK from 2004 to 2013: a population-based cohort study. *European Respiratory Journal* 2016;47(1):186-93.
53. Gayle AV, Axson EL, Bloom CI, et al. Changing causes of death for patients with chronic respiratory disease in England, 2005-2015. *Thorax* 2019;74(5):483-91.
54. Bellelli G, Chalmers JD, Sotgiu G, et al. Characterization of bronchiectasis in the elderly. *Respiratory Medicine* 2016;119:13-19.
55. Gao YH, Guan WJ, Liu SX, et al. Aetiology of bronchiectasis in adults: A systematic literature review. *Respirology* 2016;21(8):1376-83.

56. Masters NJ. Bronchiectasis. Prevalence in general practice. *BMJ* 2010;341:c4163.
57. Navaratnam V, Millett ER, Hurst JR, et al. Bronchiectasis and the risk of cardiovascular disease: a population-based study. *Thorax* 2017;72(2):161-66.
58. O'Donnell AE. Bronchiectasis update. *Current Opinion in Infectious Diseases* 2018;31(2):194-98.
59. Polverino E, Cacheris W, Spencer C, et al. Global burden of noncystic fibrosis bronchiectasis: A simple epidemiological analysis. *European Respiratory Journal Conference: European Respiratory Society Annual Congress* 2012;40(SUPPL. 56)
60. Quint JK, Smith MP. Paediatric and adult bronchiectasis: Diagnosis, disease burden and prognosis. *Respirology* 2019;24(5):413-22.
61. Roberts HJ, Hubbard R. Trends in bronchiectasis mortality in England and Wales. *Respiratory Medicine* 2010;104(7):981-5.
62. Anwar G, McDonnell M, Worthy S, et al. Phenotyping adults with non-cystic fibrosis bronchiectasis: a prospective observational cohort study. *Respiratory medicine* 2013;107(7):1001-07.
63. Dimakou K, Triantafillidou C, Toumbis M, et al. Non CF-bronchiectasis: Aetiologic approach, clinical, radiological, microbiological and functional profile in 277 patients. *Respiratory Medicine* 2016;116:1-7.
64. Buscot M, Pottier H, Marquette C-H, et al. Phenotyping adults with non-cystic fibrosis bronchiectasis: a 10-year cohort study in a French regional university hospital center. *Respiration* 2016;92(1):1-8.
65. Lonni S, Chalmers JD, Goeminne PC, et al. Etiology of non–cystic fibrosis bronchiectasis in adults and its correlation to disease severity. *Annals of the American Thoracic Society* 2015;12(12):1764-70.
66. Martínez-García MÁ, Vendrell M, Girón R, et al. The multiple faces of non–cystic fibrosis bronchiectasis. A cluster Analysis approach. *Annals of the American Thoracic Society* 2016;13(9):1468-75.
67. Säynäjäkangas O, Keistinen T, Tuuponen T, et al. Bronchiectasis in Finland: trends in hospital treatment. *Respiratory medicine* 1997;91(7):395-98.
68. Säynäjäkangas O, Keistinen T, Tuuponen T, et al. Evaluation of the incidence and age distribution of bronchiectasis from the Finnish hospital discharge register. *Central European journal of public health* 1998;6(3):235-37.
69. Auger WR, Kim NH, Trow TK. Chronic thromboembolic pulmonary hypertension. *Clinics in Chest Medicine* 2010;31(4):741-58.
70. Condliffe R, Kiely DG, Coghlan JG, et al. Survival in pulmonary hypertension registries: the importance of incident cases. *Chest* 2011;139(6):1547-48.
71. Escribano-Subias P, Blanco I, Lopez-Meseguer M, et al. Survival in pulmonary hypertension in Spain: insights from the Spanish registry. *European Respiratory Journal* 2012;40(3):596-603.
72. Fraisse A, Jais X, Schleich JM, et al. Characteristics and prospective 2-year follow-up of children with pulmonary arterial hypertension in France. *Archives of cardiovascular diseases* 2010;103(2):66-74.
73. Ling Y, Johnson MK, Kiely DG, et al. Changing demographics, epidemiology, and survival of incident pulmonary arterial hypertension: results from the pulmonary hypertension registry of the United Kingdom and Ireland. *American Journal of Respiratory & Critical Care Medicine* 2012;186(8):790-6.
74. van Loon RL, Roofthooft MT, Hillege HL, et al. Pediatric pulmonary hypertension in the Netherlands: epidemiology and characterization during the period 1991 to 2005. *Circulation* 2011;124(16):1755-64.

75. Delcroix M, Kerr K, Fedullo P. Chronic Thromboembolic Pulmonary Hypertension. Epidemiology and Risk Factors. *Annals of the American Thoracic Society* 2016;13 Suppl 3:S201-6.
76. Fernandes T, Auger W, Fedullo P. Epidemiology and risk factors for chronic thromboembolic pulmonary hypertension. *Thrombosis Research* 2018;164:145-49.
77. Franco V, Ryan JJ, McLaughlin VV. Pulmonary Hypertension in Women. *Heart Failure Clinics* 2019;15(1):137-45.
78. Jiang X, Jing ZC. Epidemiology of pulmonary arterial hypertension. *Current Hypertension Reports* 2013;15(6):638-49.
79. Jin H, Yang J, Zhang Q, et al. Epidemiology and clinical management of pulmonary hypertension in children. *Sunhwangi* 2012;42(8):513-8.
80. Klok FA, Huisman MV. Epidemiology and management of chronic thromboembolic pulmonary hypertension. *Netherlands Journal of Medicine* 2010;68(9):347-51.
81. Lau EMT, Giannoulatou E, Celermajer DS, et al. Epidemiology and treatment of pulmonary arterial hypertension. *Nature Reviews Cardiology* 2017;14(10):603-14.
82. Levine DJ. Pulmonary arterial hypertension: updates in epidemiology and evaluation of patients. *American Journal of Managed Care* 2021;27(3 Suppl):S35-S41.
83. McGoon MD, Benza RL, Escribano-Subias P, et al. Pulmonary arterial hypertension: epidemiology and registries. *Journal of the American College of Cardiology* 2013;62(25 Suppl):D51-9.
84. Medrek S, Safdar Z. Epidemiology and Pathophysiology of Chronic Thromboembolic Pulmonary Hypertension: Risk Factors and Mechanisms. *Methodist DeBakey cardiovascular journal* 2016;12(4):195-98.
85. Mocumbi AO, Thienemann F, Sliwa K. A global perspective on the epidemiology of pulmonary hypertension. *Canadian Journal of Cardiology* 2015;31(4):375-81.
86. Orem C. Epidemiology of pulmonary hypertension in the elderly. *Journal of Geriatric Cardiology* 2017;14(1):11-16.
87. Prins KW, Thenappan T. World Health Organization Group I Pulmonary Hypertension: Epidemiology and Pathophysiology. *Cardiology Clinics* 2016;34(3):363-74.
88. Swinnen K, Quarck R, Godinas L, et al. Learning from registries in pulmonary arterial hypertension: pitfalls and recommendations. *European Respiratory Review* 2019;28(154)
89. Taichman DB, Mandel J. Epidemiology of pulmonary arterial hypertension. *Clinics in Chest Medicine* 2013;34(4):619-37.
90. Thenappan T, Ryan JJ, Archer SL. Evolving epidemiology of pulmonary arterial hypertension. *American Journal of Respiratory & Critical Care Medicine* 2012;186(8):707-9.
91. Clark CB, Horn EM. Group 2 Pulmonary Hypertension: Pulmonary Venous Hypertension: Epidemiology and Pathophysiology. *Cardiology Clinics* 2016;34(3):401-11.
92. Coquoz N, Weilenmann D, Stolz D, et al. Multicentre observational screening survey for the detection of CTEPH following pulmonary embolism. *European Respiratory Journal* 2018;51(4):04.
93. Georgiopoulou VV, Kalogeropoulos AP, Borlaug BA, et al. Left ventricular dysfunction with pulmonary hypertension: Part 1: epidemiology, pathophysiology, and definitions. *Circulation: Heart Failure* 2013;6(2):344-54.
94. Gerges M, Gerges C, Pistritto AM, et al. Pulmonary Hypertension in Heart Failure. Epidemiology, Right Ventricular Function, and Survival. *American Journal of Respiratory & Critical Care Medicine* 2015;192(10):1234-46.

95. Guha A, Amione-Guerra J, Park MH. Epidemiology of Pulmonary Hypertension in Left Heart Disease. *Progress in Cardiovascular Diseases* 2016;59(1):3-10.
96. Thenappan T, Gomberg-Maitland M. Epidemiology of pulmonary hypertension and right ventricular failure in left heart failure. *Current Heart Failure Reports* 2014;11(4):428-35.
97. Cottin V, Avot D, Levy-Bachelot L, et al. Identifying chronic thromboembolic pulmonary hypertension through the French national hospital discharge database. *PLoS ONE [Electronic Resource]* 2019;14(4):e0214649.
98. Hoeper MM, Huscher D, Pittrow D. Incidence and prevalence of pulmonary arterial hypertension in Germany. *International Journal of Cardiology* 2016;203:612-3.
99. Kramm T, Wilkens H, Fuge J, et al. Incidence and characteristics of chronic thromboembolic pulmonary hypertension in Germany. *Clinical Research in Cardiology* 2018;107(7):548-53.
100. Pektas A, Pektas BM, Kula S. An epidemiological study of paediatric pulmonary hypertension in Turkey. *Cardiology in the Young* 2016;26(4):693-7.
101. Jansa P, Jarkovsky J, Al-Hiti H, et al. Epidemiology and long-term survival of pulmonary arterial hypertension in the Czech Republic: a retrospective analysis of a nationwide registry. *BMC Pulmonary Medicine* 2014;14:45.
102. Radegran G, Kjellstrom B, Ekmehag B, et al. Characteristics and survival of adult Swedish PAH and CTEPH patients 2000-2014. *Scandinavian Cardiovascular Journal* 2016;50(4):243-50.
103. Skride A, Sablinskis K, Rudzitis A, et al. First data from Latvian chronic thromboembolic pulmonary hypertension registry. *European Journal of Internal Medicine* 2016;32:e23-4.
104. Arvanitaki A, Boutsikou M, Anthi A, et al. Epidemiology and initial management of pulmonary arterial hypertension: real-world data from the Hellenic pulmOnary hyPertension rEgistry (HOPE). *Pulmonary Circulation* 2019;9(3):2045894019877157.
105. Awdish R, Cajigas H. Definition, epidemiology and registries of pulmonary hypertension. *Heart Failure Reviews* 2016;21(3):223-8.
106. Bergot E, De Leotoing L, Bendjenana H, et al. Hospital burden of pulmonary arterial hypertension in France. *PLoS ONE [Electronic Resource]* 2019;14(9):e0221211.
107. Demerouti E, Karyofyllis P, Voudris V, et al. Epidemiology and Management of Chronic Thromboembolic Pulmonary Hypertension in Greece. Real-World Data from the Hellenic Pulmonary Hypertension Registry (HOPE). *Journal of Clinical Medicine* 2021;10(19):30.
108. Dzudie A, Kengne AP, Thienemann F, et al. Predictors of hospitalisations for heart failure and mortality in patients with pulmonary hypertension associated with left heart disease: a systematic review. *BMJ Open* 2014;4(7):e004843.
109. Hoeper MM, Kramer T, Pan Z, et al. Mortality in pulmonary arterial hypertension: prediction by the 2015 European pulmonary hypertension guidelines risk stratification model. *European Respiratory Journal* 2017;50(2):08.
110. Humbert M, Sitbon O, Yaici A, et al. Survival in incident and prevalent cohorts of patients with pulmonary arterial hypertension. *European Respiratory Journal* 2010;36(3):549-55.
111. Imbalzano E, Vatrano M, Lo Gullo A, et al. Prevalence of Pulmonary Hypertension in an Unselected Community-Based Population: A Retrospective Echocardiographic Study-RES-PH Study. *Journal of Personalized Medicine* 2021;11(6):31.

112. Natali S, Palmieri M, Polidori C. Prevalence of pulmonary arterial hypertension in the Camerino area of central Italy and savings resulting from generic bosentan. *European Journal of Hospital Pharmacy Science & Practice* 2020;27(2):100-02.
113. Pepke-Zaba J, Delcroix M, Lang I, et al. Chronic thromboembolic pulmonary hypertension (CTEPH): results from an international prospective registry. *Circulation* 2011;124(18):1973-81.
114. Pfeuffer-Jovic E, Weiner S, Wilkens H, et al. Impact of the new definition of pulmonary hypertension according to world symposium of pulmonary hypertension 2018 on diagnosis of post-capillary pulmonary hypertension. *International Journal of Cardiology* 2021;335:105-10.
115. Sinan UY, Cetinarslan O, Arat Ozkan A, et al. The impact of the new World Symposium on Pulmonary Hypertension definition of pulmonary hypertension on the prevalence of pre-capillary pulmonary hypertension. *Turk Kardiyoloji Dernegi Arsivi* 2019;47(7):594-98.
116. Emmons-Bell S, Johnson C, Boon-Dooley A, et al. Prevalence, incidence, and survival of pulmonary arterial hypertension: A systematic review for the global burden of disease 2020 study. *Pulmonary Circulation* 2022;12(1):e12020.
117. Chang JL, Goldberg AN, Alt JA, et al. International consensus statement on obstructive sleep apnea. *International forum of allergy & rhinology* 2022;06 doi: https://dx.doi.org/10.1002/alr.23079
118. Krishnan S, Dhar R. Bronchiectasis Epidemiology and Risk Factors. *Current Pulmonology Reports* 2023;12(3):97-104. doi: https://dx.doi.org/10.1007/s13665-023-00317-w
119. Sezgin Y, Becel S, Kaplan AK. Comparison of COVID-19 Outcomes With Alpha-1 Antitrypsin Deficiency Prevalence in Europe: A Cross-Sectional Study. *Cureus* 2023;15(1):e34293. doi: https://dx.doi.org/10.7759/cureus.34293
120. Karki C, Hagiwara M, Chen YJ, et al. EPIDEMIOLOGY ESTIMATES FOR ALPHA-1 ANTITRYPSIN DEFICIENCY (AATD) AND AATD-ASSOCIATED LIVER DISEASE (AATD-LD) IN PEDIATRIC AND ADULT POPULATIONS BASED ON A SYSTEMATIC LITERATURE REVIEW. *Hepatology* 2022;76(Supplement 1):S1114-S15. doi: https://dx.doi.org/10.1002/hep.32697
121. Maggi S, Veronese N, Burgio M, et al. Rate of Hospitalizations and Mortality of Respiratory Syncytial Virus Infection Compared to Influenza in Older People: A Systematic Review and Meta-Analysis. *Vaccines* 2022;10(12) (no pagination) doi: https://dx.doi.org/10.3390/vaccines10122092
122. Kim C, Song S, Kim S, et al. Lung fuction and bronchiectasis incidence: Nationwide population-based cohort study. *Respirology* 2023;28(Supplement 1):144-45. doi: https://dx.doi.org/10.1111/resp.14433
123. Soudani S, Mafi A, Al Mayahi Z, et al. A Systematic Review of Influenza Epidemiology and Surveillance in the Eastern Mediterranean and North African Region. *Infectious Diseases and Therapy* 2022;11(1):15-52. doi: https://dx.doi.org/10.1007/s40121-021-00534-3
124. Li Y, Wang LL, Xie LL, et al. The epidemiological and clinical characteristics of the hospital-acquired influenza infections: A systematic review and meta-analysis. *Medicine (United States)* 2021;100(11):E25142. doi: https://dx.doi.org/10.1097/MD.0000000000025142
125. Polverino E, De Soyza A, Dimakou K, et al. The Association Between Bronchiectasis and Chronic Obstructive Pulmonary Disease: Data from the European Bronchiectasis Registry (EMBARC). *American journal of respiratory and critical care medicine* 2024;25 doi: https://dx.doi.org/10.1164/rccm.202309-1614OC

126. Landry SA, Beatty C, Thomson LDJ, et al. A review of supine position related obstructive sleep apnea: Classification, epidemiology, pathogenesis and treatment. *Sleep Medicine Reviews* 2023;72:101847. doi: https://dx.doi.org/10.1016/j.smrv.2023.101847
127. Betts JM, Weinman AL, Oliver J, et al. Influenza-associated hospitalisation and mortality rates among global Indigenous populations; a systematic review and meta-analysis. *medRxiv* 2022;30 doi: https://dx.doi.org/10.1101/2022.10.27.22281610
128. Abavisani M, Keikha M, Karbalaei M. First global report about the prevalence of multi-drug resistant Haemophilus influenzae: a systematic review and meta-analysis. *BMC Infectious Diseases* 2024;24(1):90. doi: https://dx.doi.org/10.1186/s12879-023-08930-5
129. Munteanu O, Chesov D, Rusu D, et al. Mortality related risk factors in patients with non-cystic fibrosis bronchiectasis. *European Respiratory Journal Conference: European Respiratory Society International Congress, ERS* 2022;60(Supplement 66) doi: https://dx.doi.org/10.1183/13993003.congress-2022.4014
130. Alzahrani MK. Prevalence of Obstructive Sleep Apnea in Gulf Countries-A literature review. *Journal of Pioneering Medical Sciences* 2023;12(1):24-29. doi: https://dx.doi.org/10.61091/jpms20231216
131. Stockley RA, Pye A, De Soyza J, et al. The prevalence of bronchiectasis in patients with alpha-1 antitrypsin deficiency: initial report of EARCO. *Orphanet Journal Of Rare Diseases* 2023;18(1):243. doi: https://dx.doi.org/10.1186/s13023-023-02830-2
132. Rezaei-Tavabe N, Kheiri S, Mousavi MS, et al. The Relationship between Influenza Vaccine and Hospitalization and Mortality in the Elderly: A Systematic Review and Meta-Analysis. [Persian]. *Journal of Isfahan Medical School* 2022;39(352):934-43. doi: https://dx.doi.org/10.22122/JIMS.V39I652.14356
133. Prechaporn W, Hantrakul P, Ngamjarus C, et al. Pooled prevalences of obstructive sleep apnea and heart failure: a systematic review and meta-analysis. *Heart Failure Reviews* 2024 doi: https://dx.doi.org/10.1007/s10741-024-10399-6
134. Heinzer R, Vat S, Marques-Vidal P, et al. Prevalence of sleep-disordered breathing in the general population: the HypnoLaus study. *The Lancet Respiratory Medicine* 2015;3(4):310-18.
135. Lonni S, Chalmers JD, Goeminne PC, et al. Etiology of Non-Cystic Fibrosis Bronchiectasis in Adults and Its Correlation to Disease Severity. *Annals of the American Thoracic Society* 2015;12(12):1764-70.
136. Martínez-García MÁ, Máiz L, Olveira C, et al. Spanish guidelines on treatment of bronchiectasis in adults. *Archivos de Bronconeumología (English Edition)* 2018;54(2):88-98.
137. Chalmers JD, Polverino E, Crichton ML, et al. Bronchiectasis in Europe: data on disease characteristics from the European Bronchiectasis registry (EMBARC). *The Lancet Respiratory Medicine* 2023;11(7):637-49.
138. Ringshausen FC, Rademacher J, Pink I, et al. Increasing bronchiectasis prevalence in Germany, 2009-2017: a population-based cohort study. *European Respiratory Journal* 2019;54(6):12.
